# Analyses of crop yield dynamics and the development of a multimodal neural network prediction model with G×E×M interactions

**DOI:** 10.3389/fpls.2025.1537990

**Published:** 2025-07-31

**Authors:** Saiara Samira Sajid, Zahra Khalilzadeh, Lizhi Wang, Guiping Hu

**Affiliations:** ^1^ Iowa State University, Industrial Manufacturing & Systems Engineering, Ames, IA, United States; ^2^ Oklahoma State University, Industrial Engineering & Management, Stillwater, OK, United States

**Keywords:** genotype, planting date, high-yield hybrid classification, precision farming, multimodal CNN-DNN

## Abstract

This study investigated how genotype, environment, and management (G×E×M) interactions influence yield and highlight the importance of accurate, early yield predictions for effective farm management and enhancing food security. We developed a yield prediction model capable of determining field-level outputs based on comprehensive data inputs, including genotype, spatial, temporal, environmental, and management factors. Among tested models—LASSO, Random Forest, XGBoost, single-modal CNN-DNN, and multimodal CNN-DNN—the multimodal CNN-DNN ensembled with XGBoost demonstrated superior performance. Applied to the G2F dataset covering 21 states from 2014 to 2021 across various treatments (i.e., standard, drought, irrigation, disease trials), the model excelled particularly in stable historical yield settings (RMSE 2.36 Mg/ha for standard treatment) with an overall RMSE of 2.45 Mg/ha. Additionally, we introduced an empirical tool for identifying high-yield hybrids suitable for standard and challenging conditions. Exploratory analysis confirmed that crop yields vary greatly by hybrid and location interaction and that late planting generally yields less than standard timing. Customized management strategies based on specific local and hybrid conditions are crucial for optimal yield outcomes.

## 1 Introduction

To ensure global food security, establishing a sustainable food supply chain is essential. A major factor in achieving this sustainability is enhancing crop productivity and developing precise crop prediction models. Crop yields are shaped by multiple influences, such as environmental conditions, crop hybrids, and farming practices. Gaining a deeper understanding of these factors is key to improving decision-making and improving productivity. Moreover, precise yield predictions can aid informed management decisions throughout the growing season, guiding resource allocation and ultimately helping secure a reliable future food supply.

The timing of planting is an important farm management decision that greatly influences crop yield (Swanson and Wilhelm, 1996; Lauer et al., 1999; Darby and Lauer, 2002; Anapalli et al., 2005; Williams, 2006; Van Roekel and Coulter, 2011), while the optimal planting date varies based on crop hybrid (Masud Rana et al., 2024). The effect of the planting date also changes by location due to environmental factors, with some areas experiencing a more significant impact on yield due to planting decisions (Mrubata et al., 2024). Additionally, the planting date interacts with soil properties (Bollero et al., 1996) and fertilizer application (Hankinson et al., 2015; Kaiser et al., 2016), influencing yield response. Compared to early or planting at the right time, late planting results in higher yield reduction (Moseley et al., 2024). Hence, planting time recommendations should be customized to location and crop hybrid. In this study, we examined the influence of planting dates on yield across various geographic regions and weather conditions to develop a more comprehensive understanding of their impact on crop productivity.

Selecting crop hybrids that are resilient to diverse weather conditions and soil properties is essential, particularly for areas susceptible to extreme weather events like drought or disease outbreaks. Using available tools to understand genetic variation and support crop improvement can be complex and challenging (Heffner et al., 2009; Scheben et al., 2017; Nuccio et al., 2018). Dobermann et al. (2003) empirically classified yield and discovered that yield clusters account for 60-66% of the yield variability. Additionally, Maestrini and Basso (2018) demonstrated that historical yield distributions from locations with stable data can provide accurate yield predictions. Combining these concepts, our work developed an empirical hybrid classification method to identify hybrids that are well-suited for varying weather conditions and are straightforward to use for making management decisions.

The complex interaction among meteorology, soil characteristics, management decisions, and genomic traits of hybrids with crop yield makes the prediction task challenging. Recent advances in crop genomics have paved ways to access genotype data that provides insights into how these genetic factors shape crop characteristics (Mir et al., 2019). However, incorporating genotype data, among other factors, adds to the complexity of the task, primarily due to the high dimensionality of the data.

Machine learning (ML) models have demonstrated noteworthy success in handling high-dimensional data. These models are trained based on historical data to establish the mapping function that links the input variable to the output (Medsker and Jain, 2001). Their effectiveness in predicting crop yield has been substantiated by various studies (Everingham et al., 2016; Cunha et al., 2018; Kouadio et al., 2018; Filippi et al., 2019; Shahhosseini et al., 2020, 2021a; Bali and Singla, 2021; Paudel et al., 2022; Sajid et al., 2022). These investigations highlight the ML model’s ability to comprehend intricate relationships between yield, environment, and management.

Moreover, neural networks (NN) have also proven their efficacy in capturing the intricate relationship between weather, soil, management decisions, and yield (Wang et al., 2018; Nevavuori et al., 2019; Elavarasan and Durairaj Vincent, 2020; Haque et al., 2020; Khaki et al., 2020; Shahhosseini et al., 2021b; Oikonomidis et al., 2022). For example, Shahhosseini et al. (2021b) developed an ensemble convolutional neural network (CNN)-deep neural network (DNN) architecture to predict corn yield for the US corn belt using weather, soil, and management inputs. Kolipaka and Namburu (2024) proposed a heuristic approach integrating CNN-DNN and long-short-term memory (LSTM) for yield prediction across various datasets. However, these studies have primarily focused on crop yield’s interaction with environmental and management aspects, often overlooking the incorporation of genotype data into their prediction models.

In addition to environmental (E) and management decisions (M), genotype (G) plays a pivotal role in crop yield (Beres et al., 2020; Lopez-Cruz et al., 2023; Fernandes et al., 2024). Gambin et al. (2016) discovered that G × E interactions significantly impact crop yield in a study conducted in Argentina. However, complexities arise, as genetic selection can result in traits unique to new varieties even under the same environmental conditions. The complexity further escalates when considering a wide range of environmental and management conditions (Oakey et al., 2016). Additionally, the high dimensionality of genotype data adds another layer of complexity to predicting G × E interactions.

Some studies have utilized ML and NN models alongside plant genetics to predict plant phenotype traits (Ma et al., 2018; Montesinos-López et al., 2018a; b; Crossa et al., 2019; Grinberg et al., 2020; Shook et al., 2021). Notably, Khaki and Wang (2019) employed a DNN architecture to predict yield from G × E interactions for new hybrids. A recent study by Dhaliwal and Williams (2024) demonstrated that Random Forest (RF) could predict yield for sweet corn at the field level using weather, spatial, temporal, and genetic data. ML models have demonstrated superior prediction accuracy compared to the genomic prediction tool, genomic best linear unbiased prediction (GBLUP). GBLUP lacks consideration for non-linear relations and exhibits limitations compared to ML models (Danilevicz et al., 2022). Sarzaeim and Muñoz-Arriola (2024) further combined genetic prediction models with global sensitivity analysis and highlighted weather as a key factor in yield forecasting. Moreover, Washburn et al. (2024) highlighted that diverse modeling approaches enhance predictability when considering G×E×M interactions.

In the context of using genotype data for phenotype predictions, NN have shown potential; however, their full capabilities remain largely unexplored (Danilevicz et al., 2022). Multimodal model architectures designed for multi-source data have shown improved predictability for high dimensional data (Baltrusaitis et al., 2019; Maimaitijiang et al., 2020; Shahhosseini et al., 2021b; Mia et al., 2023; Yu et al., 2024). In this study, we built a multimodal NN model to predict crop yield given weather, soil, environment, genotype, and location information.

This research utilizes the dataset provided in the G2F competition (Lima et al., 2023) and aims to examine the factors influencing agricultural productivity and predict yield by incorporating G×E×M interactions. The study includes a statistical analysis of factors impacting crop yield and the development of a prediction model. The analysis of hybrid maize yield across different environments in the first part is to understand qualitatively and quantitatively the G×E×M interactions, which motivated the design of our prediction model in the second part. The proposed multimodal NN architecture considers various elements such as weather, soil, environment, genotype, temporal and spatial factors to make field-level predictions. Additionally, this research seeks to understand the influence of these factors and to develop effective methodologies for enhancing decision-making to improve agricultural results. This research has three objectives: a) to explore historical data and identify factors influencing productivity; b) to create a hybrid selection tool for normal and extreme conditions (e.g., drought, disease outbreaks); and c) to develop a predictive model for different hybrids based on G × E × M interactions.

## 2 Materials and methods

This study aims to identify the prevailing factors impacting crop yield and assist decision-makers in agricultural production. To determine the factors influencing crop yield, an exploratory analysis was first conducted to gauge the effect of place, hybrid varieties, and approaches to management. Subsequently, to aid in hybrid selection, a tool was developed to identify hybrids that perform well even in extreme scenarios. Finally, a yield prediction model was constructed using genotype, meteorological data, soil data, and management practices to provide a reliable estimate of crop yield.

### 2.1 Data sources and preprocessing

The data used in this study were adapted from the G2F initiative (Lima et al., 2023; The Genomes To Fields Initiative, 2023), encompassing over 180,000 cornfield plots in 217 different environments. A detailed description of the data used in this study is provided in Genomes to Fields (2023). The dataset comprises six distinct files, providing trait data, metadata, soil data, weather data, genotype data, and environmental data. The primary key employed for joining across the data sources, excluding genotype data, is “Env,” a combination of location and evaluation year. The genotype data was integrated with trait data using “Hybrid” as the key index.

#### 2.1.1 Metadata

The metadata includes details on the location, irrigation, planting date, specific issues encountered during the season, and agronomic management treatments, with the primary key being “Env.” The data covers locations from 21 states with various treatment types: standard, drought, irrigated, disease trial, early planting, late planting, late stressed, and dryland. Missing values for treatment were imputed using the mode of the column (standard treatment). Issues were manually classified into six categories based on raw comments: animal attack, data issues, drought, storm, no issues, and miscellaneous. The planting date, originally in date format, was converted to the day of the year format.

#### 2.1.2 Weather data

Daily records of 16 distinct weather features were available from 2014 to 2021. Initially formatted longitudinally with the primary keys “Env” and date, the dataset was subsequently transformed into a wide format for modeling purposes. In this format, the primary key became “Env,” and the feature values for each day were transposed into columns, identified by names like “FeatureName_DayOfYear.” Furthermore, this wide dataset was subjected to additional preprocessing to aggregate the daily weather features into a weekly format.

#### 2.1.3 Soil data

The soil data contained 23 fundamental soil information, with an absence of data for the year 2014. To tackle this issue, missing values for the respective features were imputed using the mean value from other years at the same location. Despite this imputation, certain locations still lacked soil features. To resolve this, a secondary imputation was performed, replacing missing soil values with the mean value of the corresponding state.

#### 2.1.4 Environmental data

The variables in the environmental data are simulated outputs from crop simulation software named Agricultural Production Systems sIMulator (APSIM) developed by Lopez-Cruz et al. (2023). The data set included various simulated soil features such as the water supply-demand ratio, extractable soil water ratio, water movement (upwards and downwards), nitrogen leaching as NO3, soil water content, and plant-available water. These features were recorded at 10 different depths and 9 phenological stages of the crop.

The data also included simulated phenological features such as grain yield, above-ground biomass, water table, and leaf area index at 9 phenological stages of the crop. However, some simulated variables had missing values for certain location-year combinations. These missing values were imputed using the mean value of the respective feature for the same location.

#### 2.1.5 Genotype data

The initial genotype data encompassed information for 434,893 loci for 4928 hybrids. This data was preprocessed to structure the first column as the hybrid ID, while the following columns contained details on various loci. Out of these loci, the loci with missing values were dropped, which reduced the number to 45,846. It was observed that for those loci, more than 80% of hybrids had missing values. Hence, those loci were disregarded in future steps. The genotype data had values “0/0”, “0/1”, “1/0”, and “1/1” for the majority of the data. However, less than 5% of the entire data had some different values such as: “2/0”, “0/2”, “2/1”, “1/2”, “2/2”, “3/0”. A data encoding logic was applied where “0/0” was encoded as 0, “0/1” or “1/0” was encoded as 0.5, and “1/1” was encoded as 1, while any other values were encoded as 0.15. Encoding categorical values as numeric values is a well-established technique for representing DNA sequence data (Ma et al., 2018; Montesinos-López et al., 2018a; b; Khaki and Wang, 2019; Grinberg et al., 2020).

To reduce the dimensionality of genotype data, one strategy involves selecting loci with unique variations (Monroe et al., 2020). An empirical rule was applied to choose loci with diverse information. After encoding, loci where less than 4000 hybrids have a value of 0 and at least 1000 hybrids have a value of 0.5 and do not have 0.15 were selected. This selection reduced the number of loci to 4,227 while ensuring that loci do not have only zeros for all hybrids and identify loci where hybrids have different values. From these, 4,227 loci randomly, 300 were selected and used in the following analysis steps. Interestingly, the models indicated that performance remained consistent when using any randomly selected 300 loci from the full set of 4,227.

### 2.2 Data exploration

Data exploration was conducted to understand yield variations across different locations, such as states, by examining yield distribution and mean yield response. The dataset included yield values for various treatment types, including drought, irrigation, and late planting disease trials. The impact of these different treatment types on yield was analyzed and found that planting date had a significant impact on yield. Hence, the study investigated yield variations based on different planting dates.

Yield distribution across states: To understand yield distribution across states, the yield distribution for each state was visually inspected and compared. Additionally, new features were created to determine the mean yield for each hybrid and state combination, which were then used in building the yield prediction model.

Yield distribution across treatment types: Another focus was on understanding how yield varies across different treatment types. For example, to examine how yield distribution changes from standard conditions to drought conditions, the yield distributions for various treatment types were compared, along with the mean yield for each treatment type.

Impact of planting date on yield: The planting date is one of the vital decisions in plant management (Dobor et al., 2016). While optimum planting dates vary based on spatial location (Thorburn et al., 2017), and in some locations, late planting can reduce crop yield (Tsimba et al., 2013). In this research the impact of planting date was analyzed across 20 states and considering more than 4000 crop hybrids. The impact of planting date on yield was analyzed by the following five-step:

*Step 1:* Compare the yield distribution for different planting dates across states.
*Step 2:* Identify planting date and state combinations corresponding to lower or higher yield.
*Step 3:* Identify hybrids with lower or higher yields.
*Step 4:* Compare those hybrids’ standard planting time with planting time in other treatments.
*Step 5:* Compare those hybrids’ yield distribution in standard planting time versus different planting time (early or late).

First, yield distributions for different planting dates were visualized and compared in terms of mean and standard deviation. The second step of the analysis revealed that the “late planting” treatment resulted in lower yields in Texas. In the third step, hybrids planted during the late-planting period were identified, and their planting dates in standard, drought, and irrigation treatments were compared in the later step. Finally, yield distributions at the hybrid level were compared for standard and late planting treatments to understand the impact of planting date on hybrids.

### 2.3 High-yield hybrid identification

**Figure 1:**
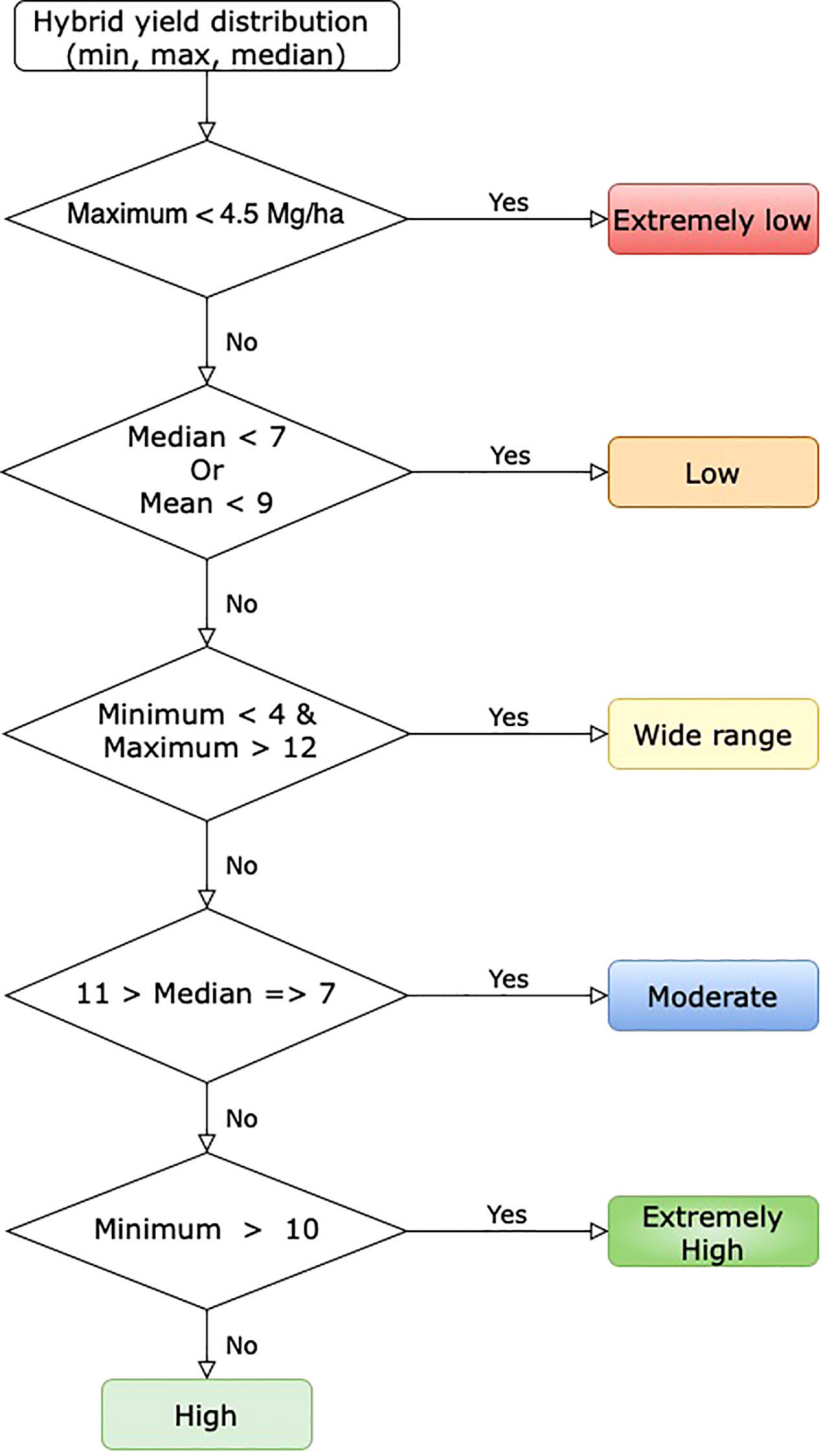
Empirical hybrid classification rule based on yield distribution.

We developed a tool to identify hybrids with higher yields across various treatment types. The tool aids in selecting high-yield hybrids for specific scenarios. To build this tool, the yield distribution of each hybrid was analyzed in the first step. In the next step, an empirical classification logic was established using the maximum, minimum, median, and mean yields of each hybrid (**Figure 1**). In the last step, these classification rules were used to create a heatmap, where each box and its color represent a hybrid’s yield performance for a treatment type. High-yield hybrids can be identified by visually locating those in the high-yield group across all treatments.

### 2.4 Predictive modeling

A yield prediction model was developed by integrating genotype, environment, and management data. Various modeling approaches were explored, including linear models, tree-based models, and neural networks. To enhance model performance, new features were created before building the prediction model. After evaluating the performance of different models, an ensemble of tree-based models and neural networks demonstrated the best performance. The model was trained on data from the year 2014 to 2020, and 2021 served as an independent testing set.

This section outlines the methodology for the proposed prediction model across three subsections. Subsection 2.4.1 introduces additional features engineered to enhance model performance, derived from patterns and insights identified during the earlier exploratory analysis. Subsection 2.4.2 details the baseline models used for performance benchmarking. Subsection 2.4.3 provides a detailed explanation of the hybrid CNN-DNN + XGBoost model, describing how heterogeneous data sources (genotype, environment, and management) are processed and integrated through separate input channels to effectively capture complex interactions. The section systematically covers all steps leading up to prediction, including data processing, feature engineering, and model comparison.

#### 2.4.1 Feature engineering

##### 2.4.1.1 State and hybrid-level features

To account for how yield may vary by hybrid and state, features were generated for each hybrid-state combination by calculating the mean, maximum, and minimum yields using data up to 2020, the final year of the training set. For the test year 2021, there were many new hybrid-state combinations. Since this data was unavailable in the training set, missing values were imputed by averaging the mean and mode of the column grouped by state. These features were then incorporated into the prediction model.

##### 2.4.1.2 Hybrid level features

A set of features was created at the hybrid level to help the model understand yield distribution variations by hybrid. For each hybrid, the mean, maximum, and minimum yields during training were calculated and used as features. Since some hybrids appeared only once or twice in the training data, their mean, maximum, and minimum yields did not adequately represent their yield distribution. To address this, two additional features were created to represent the mean yield of both hybrid parents.

##### 2.4.1.3 Yield trend feature

The yield trend feature was generated at the state level due to incomplete yearly yield data for individual fields. A univariate linear regression model was applied to each state’s yield values from the training years. The yield trend for year n was then calculated using the regression coefficients (Equation 1).


(1) $$yield\;trend_{sn}=a_{so}+a_{s1}\left(n\right)$$

Where,

${\text{yield trend}}_{\text{sn}}=\text{yield trend for state s in year n}$ .

${\text{a}}_{\text{so}}=\text{intercept for state s}$ .

${\text{a}}_{\text{s}1}=\text{slope for state s}$ .

#### 2.4.2 Base learning models

##### 2.4.2.1 LASSO

Lasso is a linear regression variant with an L1 regularization feature in the model’s loss function, which helps to build a model with only important features by assigning zero to less important ones, thus avoiding overfitting (Tibshiranit, 1996; James et al., 2013). In this research, Lasso was implemented using the scikit-learn package (Buitinck et al., 2013) in Python to make yield predictions. The optimal L1 regularization penalty was set to 0.05 based on a grid search.

##### 2.4.2.2 Random Forest regressor

Random Forest (RF) is an ensemble tree-based model that creates multiple uncorrelated trees using a bootstrap resampling method (Breiman, 2001; Cutler et al., 2007). Each tree is constructed on a subsample of data and a subset of input features (Brown, 2017), continuing this process until all trees are formed (Cutler et al., 2007). For regression, the final prediction is the average of these trees. This research implemented the RF model using the RandomForestRegressor from the scikit-learn package (Buitinck et al., 2013). Hyperparameters were selected through a grid search, while default values were used for other parameters (**Table 1**).

**Table 1:** Optimum hyperparater values used for RF Regressor.

Hyperparameter	Value
Maximum depth:	15
Maximum number of features:	Square-root of total number of features
Minimum sample required to split:	15
Number of trees:	400

##### 2.4.2.3 XGBoost regressor

The gradient-boosting tree-based model, XGBoost, was employed for yield prediction. This model learns sequentially from weak learners, with trees generated using the “exact” method (Chen and Guestrin, 2016). The final prediction is made by aggregating all weak base learners. Optimal hyperparameters for the best-performing model were found using a grid search method (**Table 2**).

**Table 2:** Optimum hyperparater values used for XGBoost Regressor.

Hyperparameter	Value
Maximum depth:	5
Eta (step size shrinkage):	0.05
Sub sample:	0.75
Number of trees:	4000

##### 2.4.2.4 Single modal CNN-DNN

**Figure 2:**
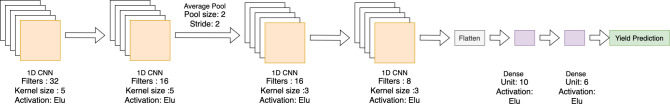
Single modal CNN-DNN architecture with four 1D CNN layers followed by two fully connected dense layers.

A single-modal CNN-DNN model was designed to predict yield, combining all numerical data sources and encoding categorical features (**Figure 2**). These inputs were passed through an 8-layer CNN-DNN model. The input layer was followed by two 1D CNN layers with 32 and 16 filters, respectively, both having a kernel size of 5 and an average pooling layer. This was connected to two more 1D CNN layers, followed by a flattening layer and three fully connected layers. The final fully connected layer served as the output layer for yield prediction (**Table 3**).

**Table 3:** Hyperparater values used for Single modal CNN-DNN.

Hyperparameter	Value
Maximum depth:	3
Eta (step size shrinkage):	0.01
Learning rate:	0.01
Sub sample:	0.15
Colsample_bytree:	0.15
Number of trees:	400

#### 2.4.3 Proposed CNN-DNN with XGBoost model

The proposed CNN-DNN with the XGBoost model is an ensemble model that combines CNN-DNN and XGBoost models. The XGBoost model was trained on metadata and features representing mean yield at the state level, hybrid level, and hybrid-state yield combinations. Meanwhile, the CNN-DNN model was trained on weather, soil, environmental (from APSIM), genotype data, and metadata.

**Figure 3:**
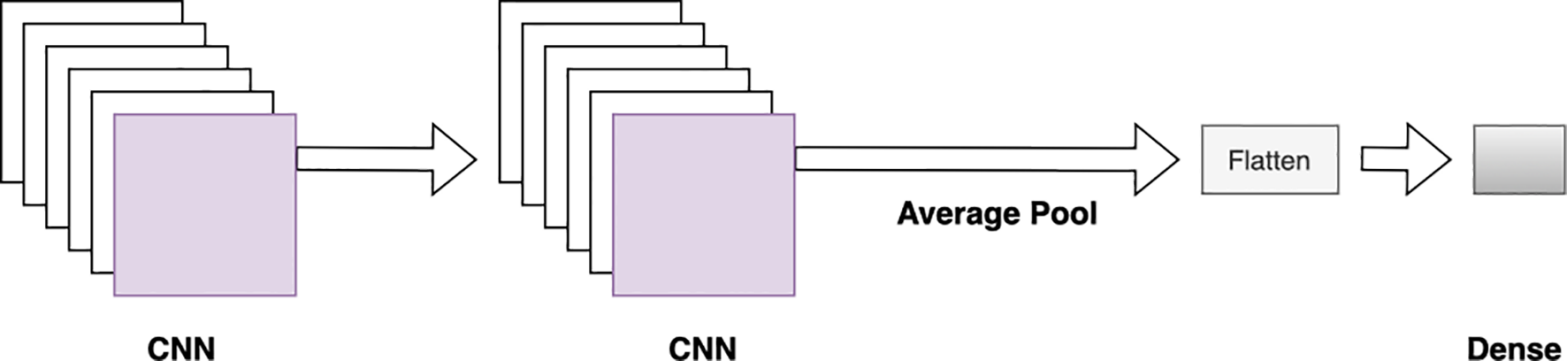
CNN block architecture. Two CNN layers are followed by an average pooling, flattening, and fully connected layer.

For the weather block, the 16 district weather features from weeks 1 to 48 were converted into a 3D array. The input layer for the weather block had a shape of (n×48×16) to maintain the time sequence, which then passed through two 2D CNN layers, followed by average pooling, flattening, and dense layers (**Figure 3**).

**Figure 4:**
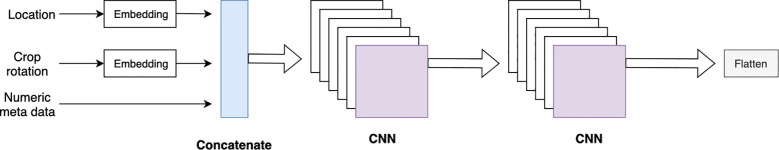
CNN block with embedding layers. The embedding layers and numeric metadata are concatenated, which is then followed by two 1D CNN layers and a flattened layer.

The 23 soil features and 300 genotype features lacked any specific order, so the soil and genotype blocks used 1D-CNN layers. The metadata included categorical features, such as “field location” and “crop rotation,” which were incorporated into the CNN-DNN model and processed through two embedding layers. These embedding layers, along with other numerical features, were then passed through two 1D-CNN layers followed by a flattening layer (**Figure 4**).

**Figure 5:**
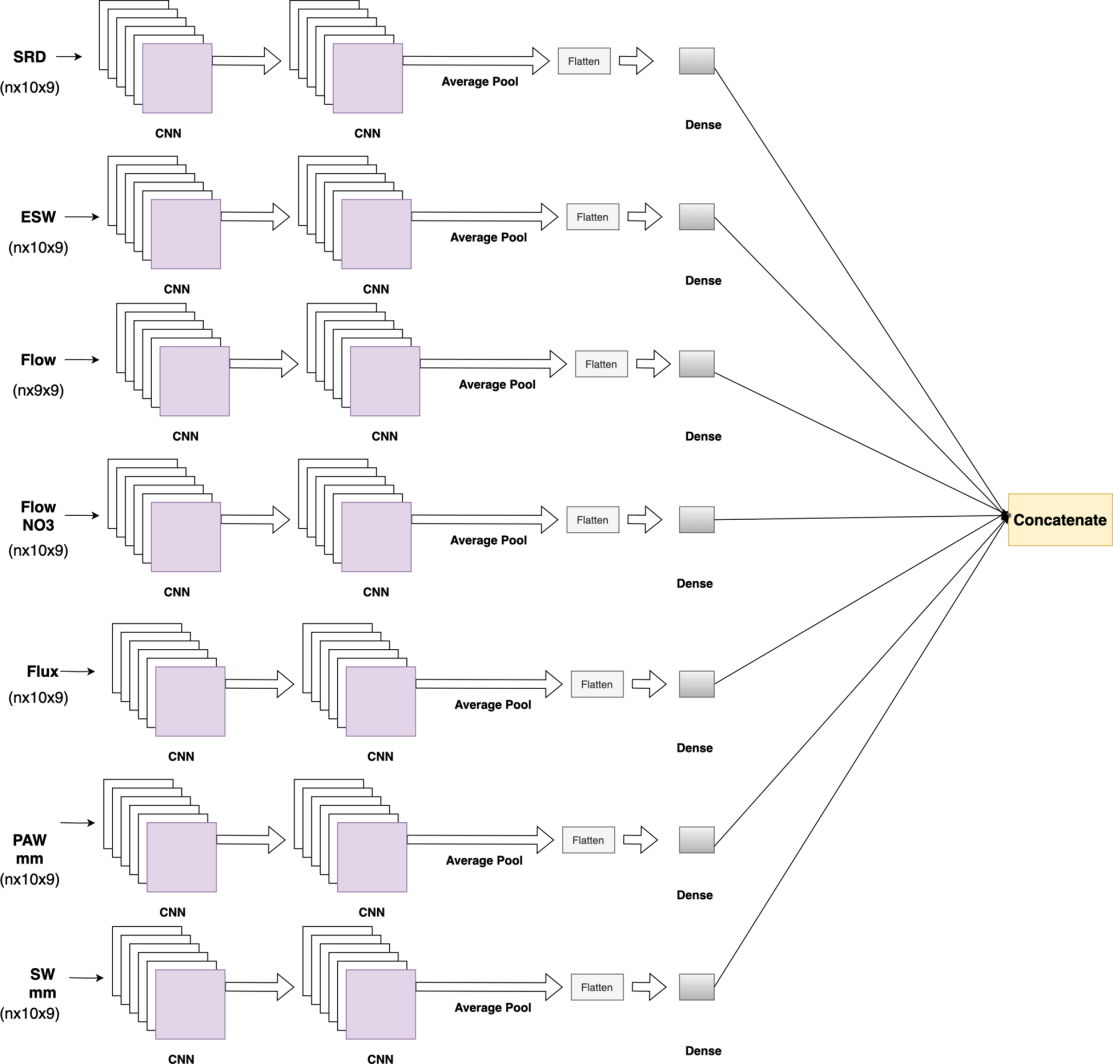
Environmental soil feature block, where each soil feature at different soil depths and phenological stages passes through 2D CNN layers. At the end, outputs from the soil features are concatenated.

Environmental features included soil-related data at 10 depths and 9 phenological stages, except for the Flow feature, which had values at 9 depths and 9 phenological stages. These soil features were reshaped to (n×10×9), while the Flow feature values were reshaped to (n×9×9). Each feature passed through two 2D CNN layers, followed by average pooling, flattening, and dense layers. The environmental soil block comprised 7 separate CNN blocks, which were concatenated to form the output of the environmental soil block (**Figure 5**).

**Figure 6:**
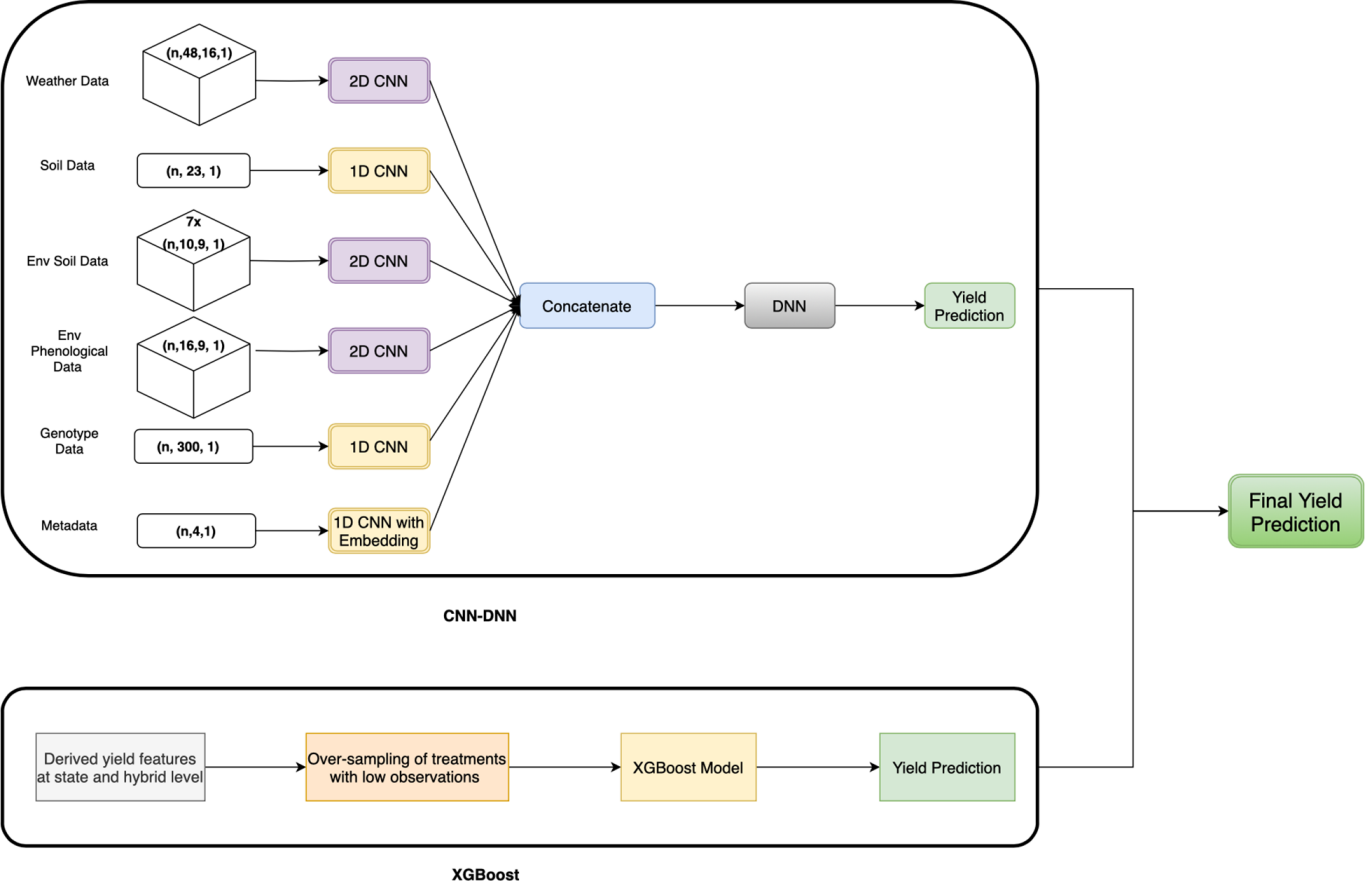
Proposed CNN-DNN framework with XGBoost model. The weather data, environmental soil data (from APSIM), and environmental phenological data (from APSIM) flow through separate 1D CNN blocks, while the soil data and genotype data pass through 1D CNN blocks. The metadata passes through the 1D CNN layer with embedding layers. The output of all the blocks is concatenated, followed by the DNN layer and the output layer. This yield prediction is ensembled with the yield prediction from XGBoost, trained on hybrid and state-level yield features only.

The CNN-DNN model includes six separate CNN blocks for different types of input data, with five blocks having similar architectures. Each block contains two CNN layers followed by average pooling, flattening, and dense layers (**Figure 3**). Depending on the input features, some blocks used 1D CNN layers, while others used 2D CNN layers (**Figure 6**).

The output of all six blocks was concatenated and passed through two fully connected layers with L1-regularization to avoid over-fitting on training data (**Figure 6**). The number of kernels, filters, and activation functions used in each layer is detailed in **Table 1** of **Supplementary Material**

The proposed architecture employed the exponential linear unit (ELU) activation function for all layers except the output layer. The choice of ELU over the rectified linear unit (ReLU) was based on its advantage in allowing negative values, thereby causing the unit activations to approach zero with reduced computational complexity (Clevert et al., 2015). Following is the mathematical formula used in ELU activation

ELU with α>0,


g(x)=x                   if x>0

g(x)= α(exp(x)−1)    if x≤0

XGBoost with oversampling: To improve predictability for drought, disease trials, or late planting time, oversampling was applied to the training set because there were fewer observations for these treatments compared to the standard treatment. For each treatment type, excluding standard and late planting, 4,000 samples were randomly selected with replacement. As the training data had no treatment corresponding to the late planting scenario, an additional 1,000 samples were selected from each treatment type, renaming the treatment type to late planting, resulting in an additional 25,000 observations along with the real data. This oversampled data was used to build the prediction model, using only metadata, hybrid-level, and state-hybrid-level yield features (**Figure 6**). The XGBoost model was employed for prediction with hyperparameters selected from a grid search approach.

The predictions from the XGBoost model and the CNN-DNN model were combined using a weighted average. To determine the optimal weights for combining CNN-DNN and XGBoost, we performed a grid search by varying the weights in 0.1 increments. The optimal weights were assigned as 0.1 to the XGBoost model predictions and 0.9 to the CNN-DNN model predictions.

All models, baseline models, and proposed CNN-DNN with and without XGBoost, were evaluated in terms of multiple metrics. Those are root mean squared error (RMSE), relative root mean squared error (RRMSE), mean absolute percentage error (MAPE) and Pearson correlation. The model with the lowest RMSE, RRMSE, MAPE and higher Pearson correlation was selected for prediction.

RMSE is calculated utilizing Equation 2.


(2) $$\text{RMSE}=\;\sqrt{\frac{\sum_{i=1}^{n}{\left(y_{i}-{\hat{y}}_{i}\right)}^{2}}{n}}$$

RRMSE is calculated using Equation 3.


(3) $$\text{RRMSE}=\;\frac{\text{RMSE}}{m_{y}}$$

MAPE is calculated by Equation 4.


(4) $$\text{MAPE}=\;\frac{\sum_{i=1}^{n}|{\text{y}}_{\text{i}}-{\hat{y}}_{\text{i}}|}{m_{y}}$$

Pearson correlation is calculated through Equation 5.


(5) $$\text{Correlation}=\frac{\sum \left(y_{i}-m_{y})({\hat{y}}_{i}-m_{\hat{y}}\right)}{\sqrt{\sum {\left(y_{i}-m_{y}\right)}^{2}\sum {\left({\hat{y}}_{i}-m_{\hat{y}}\right)}^{2}}}$$

where,

${\text{y}}_{\text{i}}\text{ is the ith obsevarion of the response variable}$ .

${\hat{y}}_{\text{i}}\text{ is the ith prediction of the response variable}$ .

${\text{m}}_{\text{y}}\text{ mean of the obsevred response variable}$ .

${\text{m}}_{\hat{\text{y}}}\text{ mean of the prediction of response variable}$ .

## 3 Results and analyses

This section presents the findings from analyzing factors impacting crop yields, including location, hybrids, and management decisions. Additionally, it includes the hybrid classification results using the empirical rule-based tool for identifying high-yield hybrids in different conditions (i.e., drought, disease trail). Finally, the results and performance evaluations of the predictive models are provided.

### 3.1 Exploratory analysis

Exploratory analysis was conducted to assess the impact of G×E×M factors on crop yield and identify those with the greatest influence. The analysis specifically examined the effects of location, management practices, and hybrid type on crop yield.

**Figure 7:**
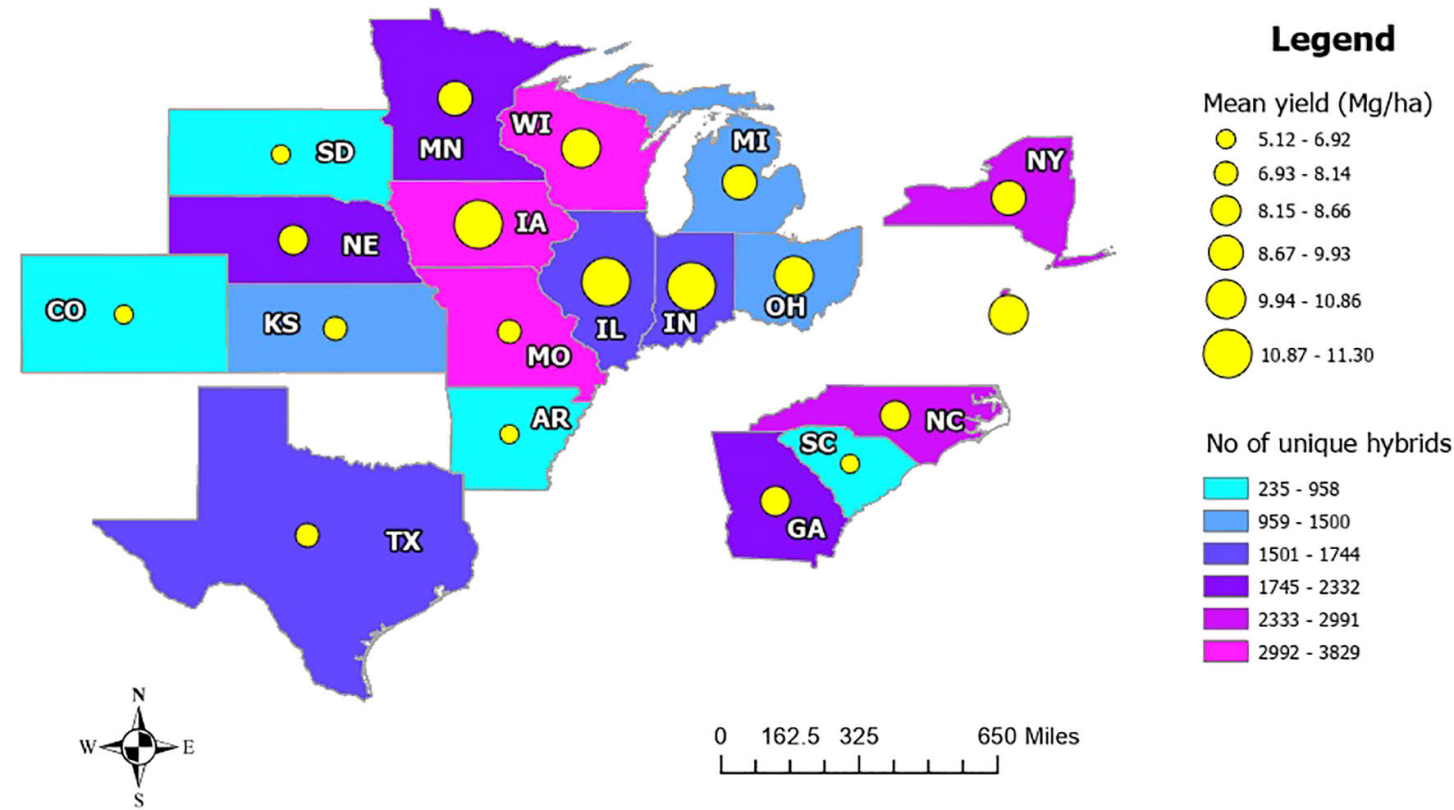
Mean yield variation in different states along with different numbers of hybrids planted. Yellow circles represent mean yield, while a larger circle corresponds to higher yield. The colors represent the number of unique hybrids planted, where sky blue is for lower hybrid variety.

*Yield distribution across states:* A comparison of mean yield and the number of unique hybrids planted at the state level revealed that areas with lower mean yields tend to have fewer hybrid varieties. For instance, states like Colorado, South Dakota, Arkansas, and South Carolina have 235 to 958 hybrid varieties, with mean yield ranging from 5.12 to 6.92 Mg/ha. In contrast, states such as Iowa, Illinois, and Indiana have mean yield ranging from 10.87 to 11.30 Mg/ha, with more than 1,500 different hybrids planted. This suggests that states with a higher number of different hybrids tend to have higher average yield (**Figure 7**).

Further analysis of yield distribution at the state level revealed significant variation among states. For instance, Iowa, Illinois, Indiana, and Missouri have unimodal yield distributions, though the mode varies. Conversely, other states, Arkansas and Kansas display a bimodal distribution, indicating that yield distribution varies based on demographic locations (**Supplementary Material Figure 1**).

**Figure 8:**
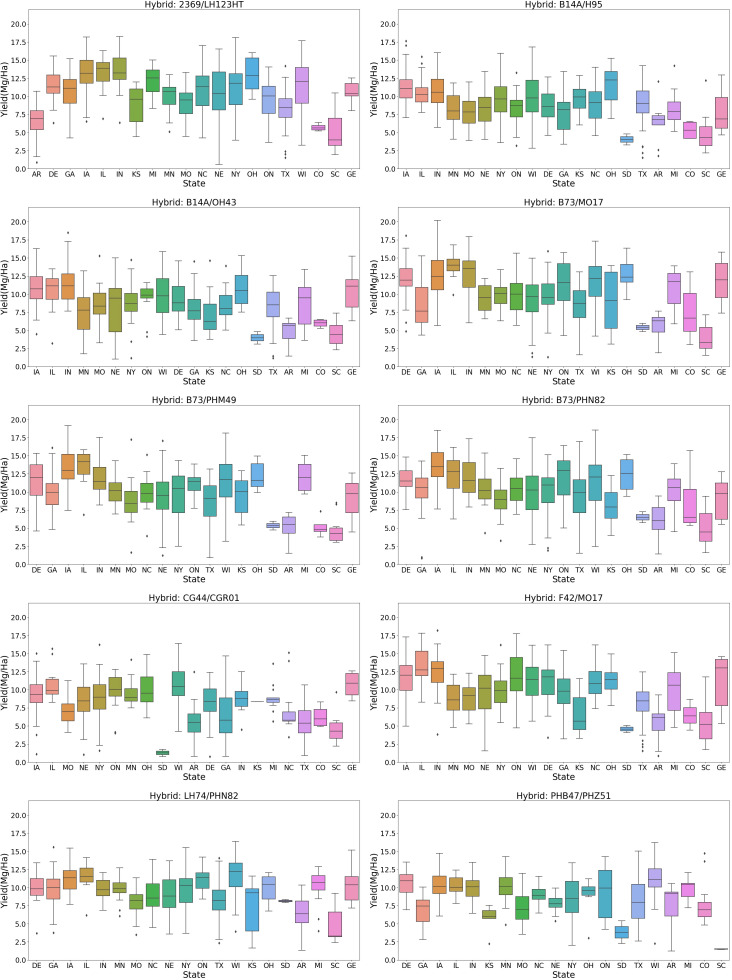
Variability in crop yield distribution across locations for the same hybrid.

Further analysis compared hybrids planted across multiple states (**Figure 8**). The yield distribution comparison revealed that a hybrid with a high yield in one state might have a lower yield in another. For example, the hybrid “2369/LH123HT” has its 25th percentile yield above 10 Mg/ha in states like Delaware, Iowa, Illinois, Indiana, Michigan, Ohio, Wisconsin, and Georgia. However, in Colorado, South Carolina, and Arkansas, its 75th percentile yield is less than 8 Mg/ha. Conversely, hybrids like “B73/MO17” and “B73/PHN82” have higher mean yields in Colorado compared to “2369/LH123HT.” This indicates that a hybrid’s high yield in one location does not guarantee similar performance elsewhere, highlighting the crucial role of hybrid-environment interactions in crop yield.

**Figure 9:**
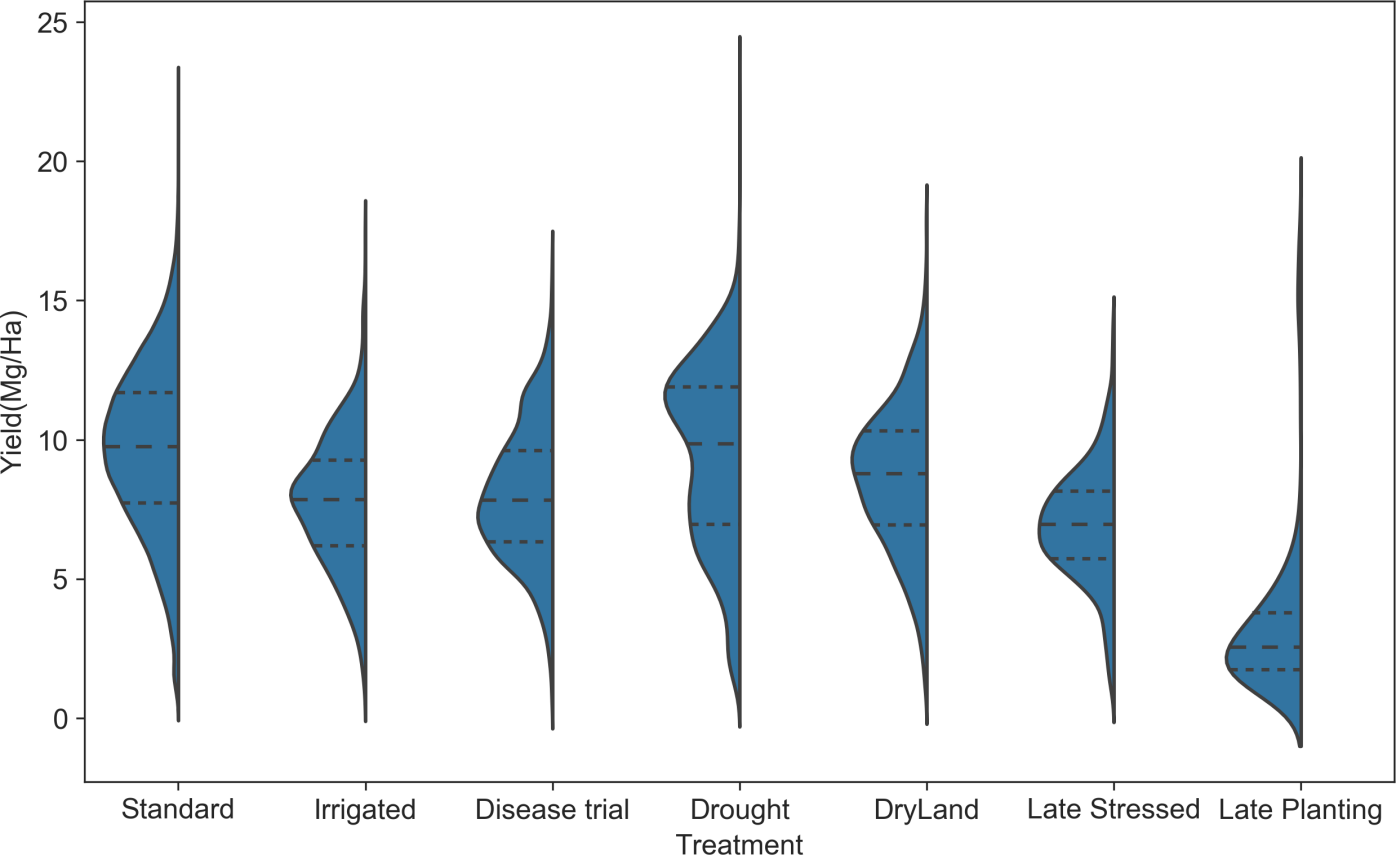
Yield distribution for different treatment types. The standard condition has a distribution with the highest mean, and late planting treatment has the lowest mean.

*Yield distribution across treatment types:* The yield distribution varies across different treatments (**Figure 9**). Standard treatment has the highest mean yield (~10 Mg/ha) and higher values at the 25th and 75th percentiles. Treatments such as irrigation, disease trials, dry land, and late stress show lower mean yield values than standard treatment, indicating reduced yield under these agronomic conditions. The yield distribution for drought conditions is bimodal, with modes at 6 Mg/ha and 12 Mg/ha, indicating that yield in drought can be moderate or lower. While, in Texas, the late planting treatment caused the lowest yield, highlighting the significant impact of planting decisions on yield. This observation prompted a detailed analysis of the planting date’s effect on crop yield.

*Impact of planting date on yield:* The five steps mentioned in section 2.2 to analyze the impact of planting date were applied to the entire data. The analysis starts with identifying the difference in yield distribution for different planting dates and expanding to yield distribution for hybrid and planting date combinations.

Step 1- Yield distribution comparison for different planting dates across states:

The impact of planting dates on yield varies significantly across states (**Supplementary Material Figure 2**). For instance, in Georgia, planting in mid-May (day 132 of the year) results in a higher yield distribution mean, while in South Carolina, planting after April (day 120) yields a lower mean. States in close geographical proximity often have similar planting dates. For example, Iowa, Illinois, and Nebraska typically plant from late April (day 114) to the end of May (day 150). Conversely, in warmer states like Texas, planting dates range from early March to early May (day 60 to day 128). Therefore, what is considered early planting in colder regions might be late for warmer locations, indicating that the impact of planting dates on crop yield varies by location.

Steps 2 & 3- Identify planting date, state combinations, and hybrids corresponding to lower yield:

In Texas, planting around mid-April (day 99) resulted in lower yield distribution and was categorized as a late planting condition. Further analysis was conducted at the hybrid level, selecting 21 hybrids planted late in Texas with yield data across all treatments. These hybrid IDs are encoded in letters and, in the rest of the paper, will be presented using these letters as given in **Table 2** of **Supplementary Materials**.

**Figure 10:**
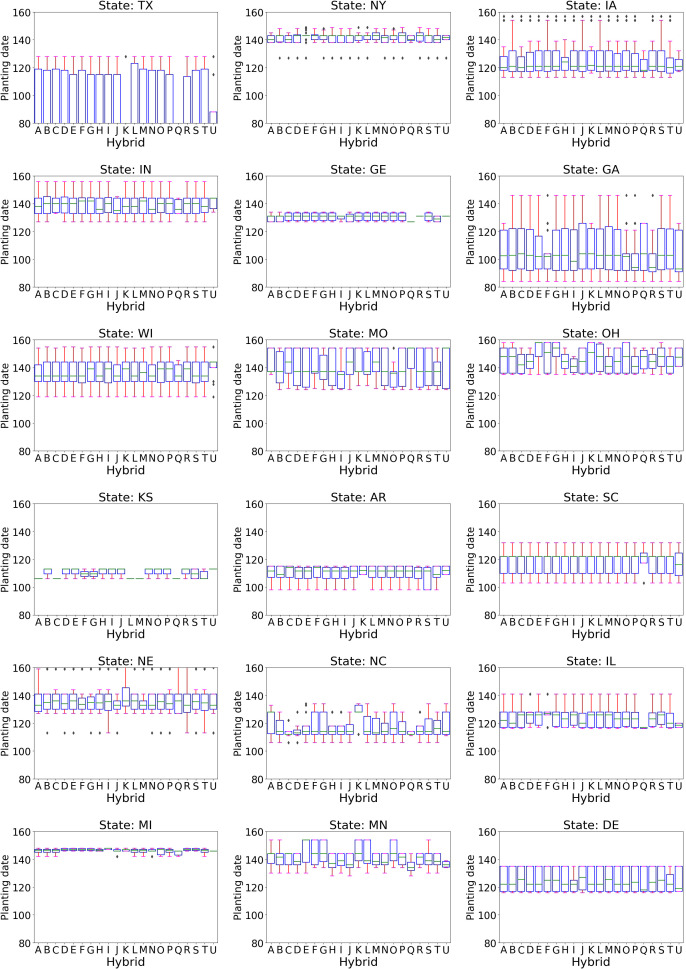
The boxplot of planting dates in different states in standard condition for 21 hybrids planted during the late planting scenario in Texas. The planting dates vary across states.

Step 4 – Comparing selected hybrids’ planting time in a standard scenario with other treatments:

The standard treatment planting date distribution revealed that these hybrids are typically planted in Texas in the third week of March (day 80). For colder locations, the planting dates for these hybrids ranged from mid-April to the end of May (days 107 to 150) (**Figure 10**). These dates were considered standard based on the local weather conditions. The planting periods for these hybrids were also examined for other treatments: early May to late May (days 125 to 150) for drought treatment, the last two weeks of May (days 140 to 154) for disease trials, and mid-March to mid-May for dryland (**Supplementary Material Figure 3**). These planting windows for other treatments in various locations align with the standard planting times. The mid-April planting date in Texas is outside the standard planting window for these hybrids for that location.

**Figure 11:**
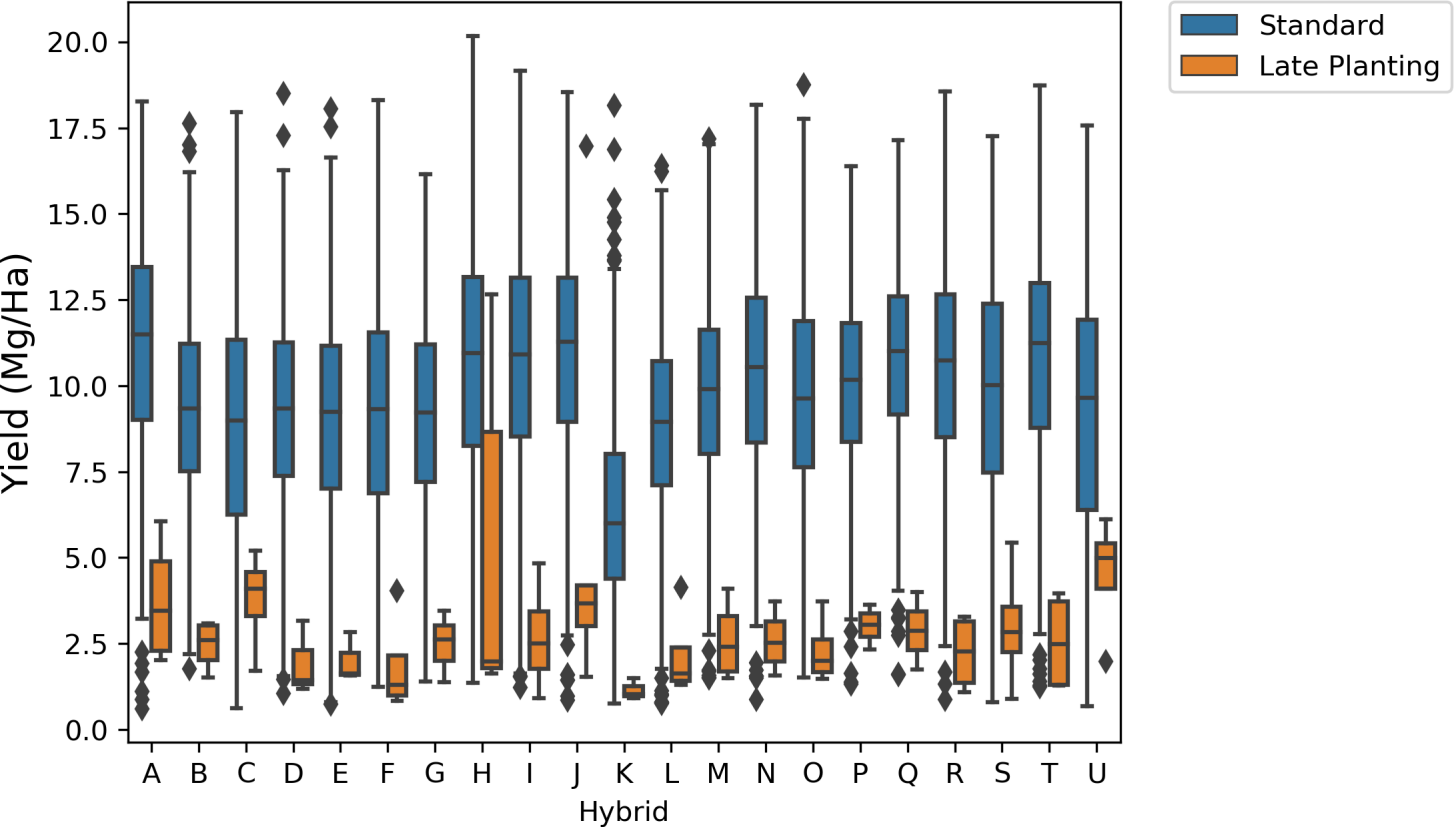
Comparison of yield distribution for hybrids in standard and late planting times. Late planting results in lower yield compared to standard planting time.

Step 5- Compare hybrids’ yield distribution in standard planting time versus late planting time:

Comparing these hybrids’ yield distribution in standard treatment and late planting time revealed that yields were lower when planting dates differed from the standard time (**Figure 11**). All 21 hybrids exhibit substantially lower yield distribution when planted late in the season.

### 3.2 High-yield hybrid identification tool

**Figure 12:**
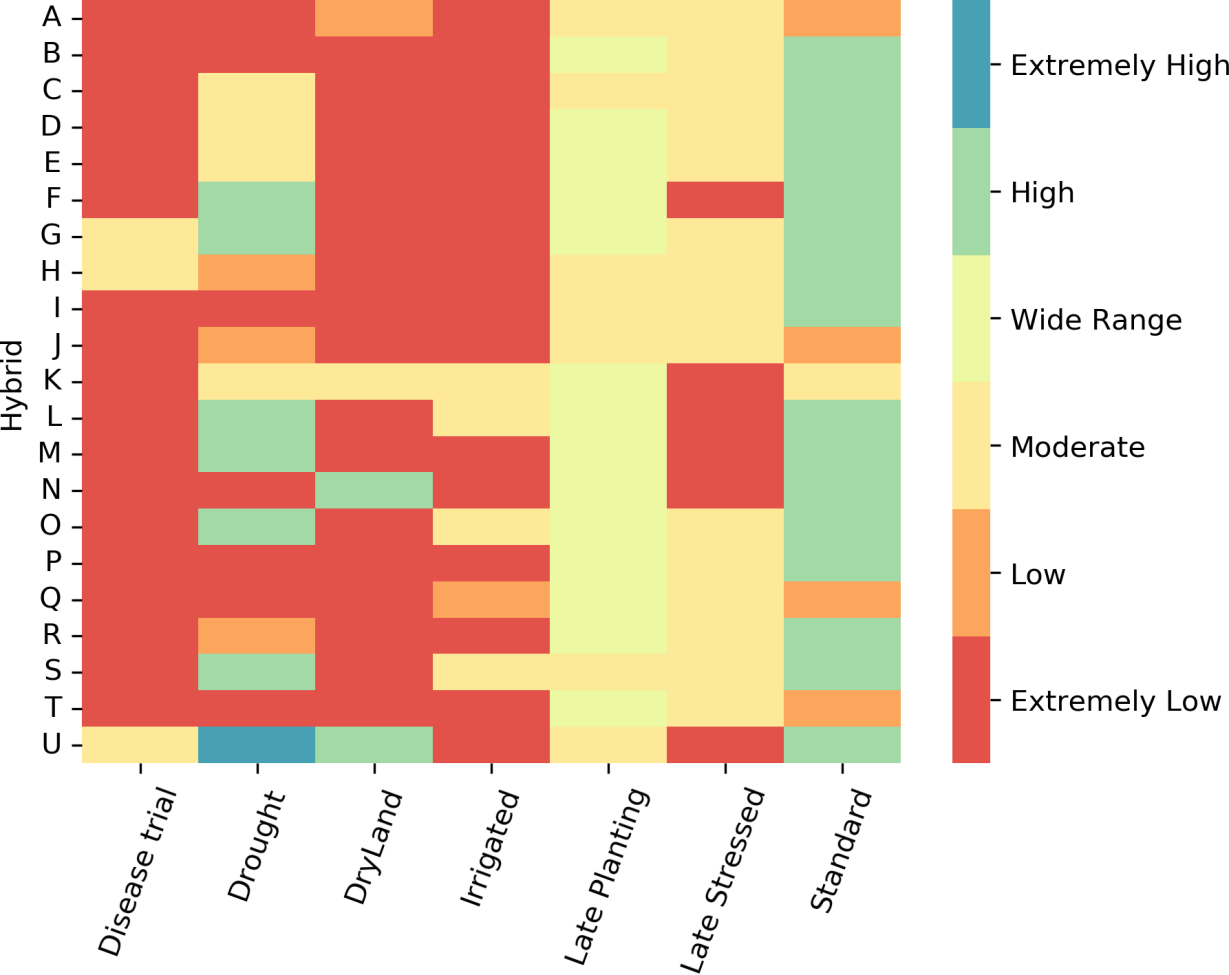
Empirical rule-based tool to identify hybrids with high yield for different treatments. Each column represents a different treatment, and each row represents a hybrid. Different color corresponds to yield class.

The visual tool for identifying hybrids is color-coded based on their yield categories: extremely low, low, moderate, wide range, high, and extremely high. These classifications are derived from the yield distribution rules outlined in section 2.3 that use empirical classification based on yield distribution (**Figure 1**). A prototype of the visual tool includes 21 hybrids commonly found across various treatment types. Red indicates hybrids with extremely low yields in all treatment combinations, while blue represents hybrids with extremely high yields (**Figure 12**).

This tool aids in selecting high-yield hybrids, particularly for locations prone to drought, dryland, and disease. For example, hybrids such as TX7777/LH195 (**U**), PHW52/PHN82 (**T**), F42/OH43 (**O**), F42/H95 (**M**), CG444/CGR01 (**L**), B37/MO17 (**H**), and B37/OH43 (**G**), which correspond to high yield categories in both standard and drought conditions, are ideal choices. It helps identify hybrids with moderate to high yields across most treatment types. For instance, the hybrid TX777/LH195(A) is suitable for scenarios involving disease attacks, drought, and standard conditions. The tool can be modified to suggest hybrids suited for specific treatment types for a broader selection.

### 3.3 Yield prediction and analysis

The prediction model was trained on data from 2014 to 2020, including yield scenarios from 21 states. The training data had a mean yield of 9.44 Mg/ha, with a standard deviation of 2.99 Mg/ha, and ranged from a minimum of 0.5 Mg/ha to a maximum of 23.27 Mg/ha. This demonstrates a wide range of yield values across different states in the training data. The prediction models were evaluated using yield values from 2021, which had a mean of 10.04 Mg/ha. The yield distribution for the test year differed from the training years, with a standard deviation of 2.78 Mg/ha and yields ranging from 0.58 Mg/ha to 18.77 Mg/ha.

**Figure 13:**
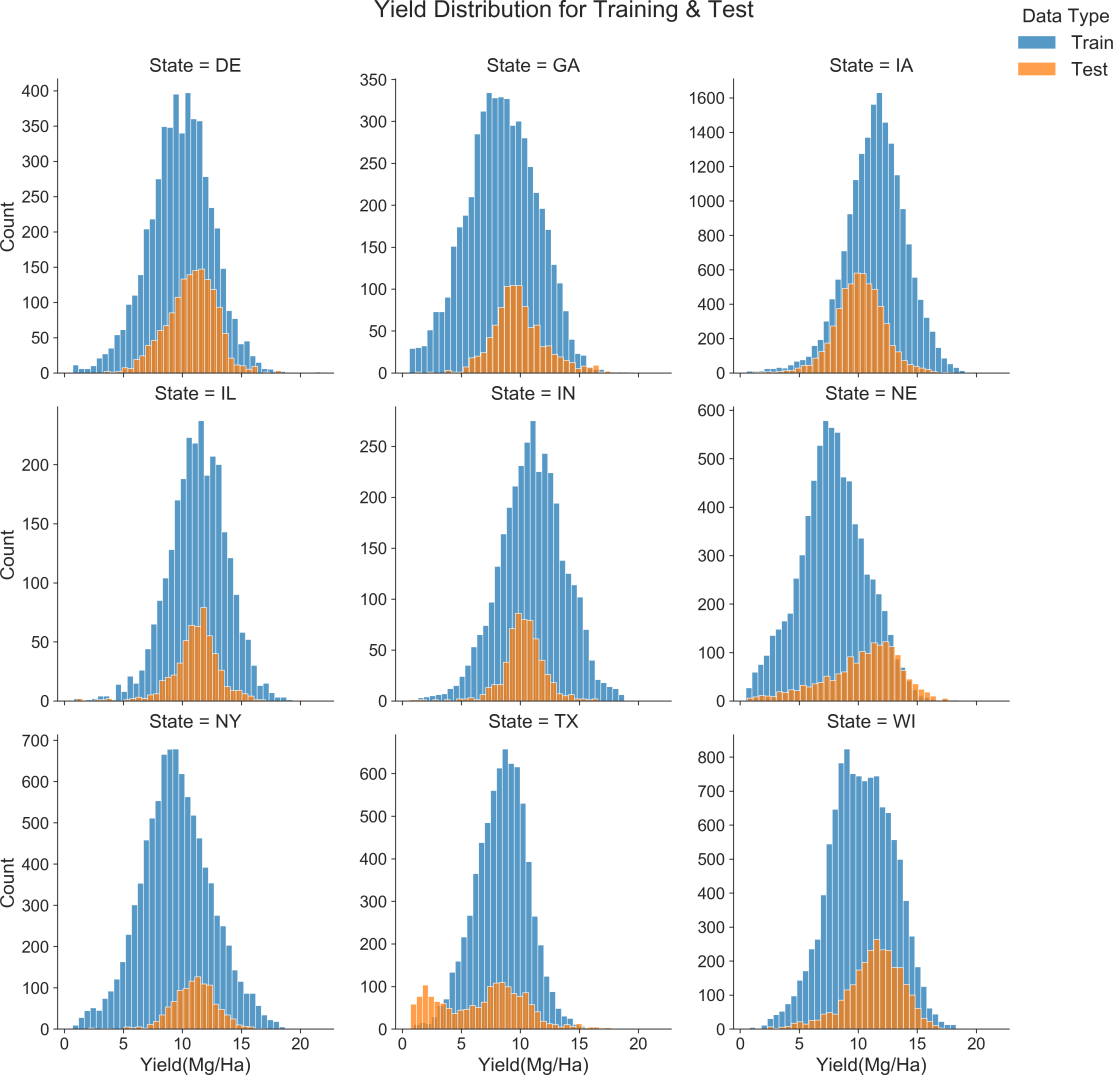
Comparison of yield distribution for training (2014-2020) and test (2021) year.

**Figure 13** compares the yield distributions across training and testing sets for various U.S. states, including Delaware (DE), Georgia (GA), Iowa (IA), Illinois (IL), Indiana (IN), Nebraska (NE), New York (NY), Texas (TX) and Wisconsin (WI). The analysis indicated significant discrepancies between the yields in training and testing periods at the state level. For example, in Iowa, the training set’s average yield was 11.5 Mg/ha, which decreased to 10.12 Mg/ha during the test year. Texas exhibited a reduction from 8.4 Mg/ha in the training set to 6.8 Mg/ha in the test year, with the test year also showing a bimodal distribution. Conversely, in New York and Nebraska, the test sets recorded higher mean yields (NY: 11 Mg/ha; NE: 10.4 Mg/ha) compared to the training sets (NY: 9.5 Mg/ha; NE: 7.8 Mg/ha), highlighting regional variations in yield consistency between the periods under review.

**Figure 14:**
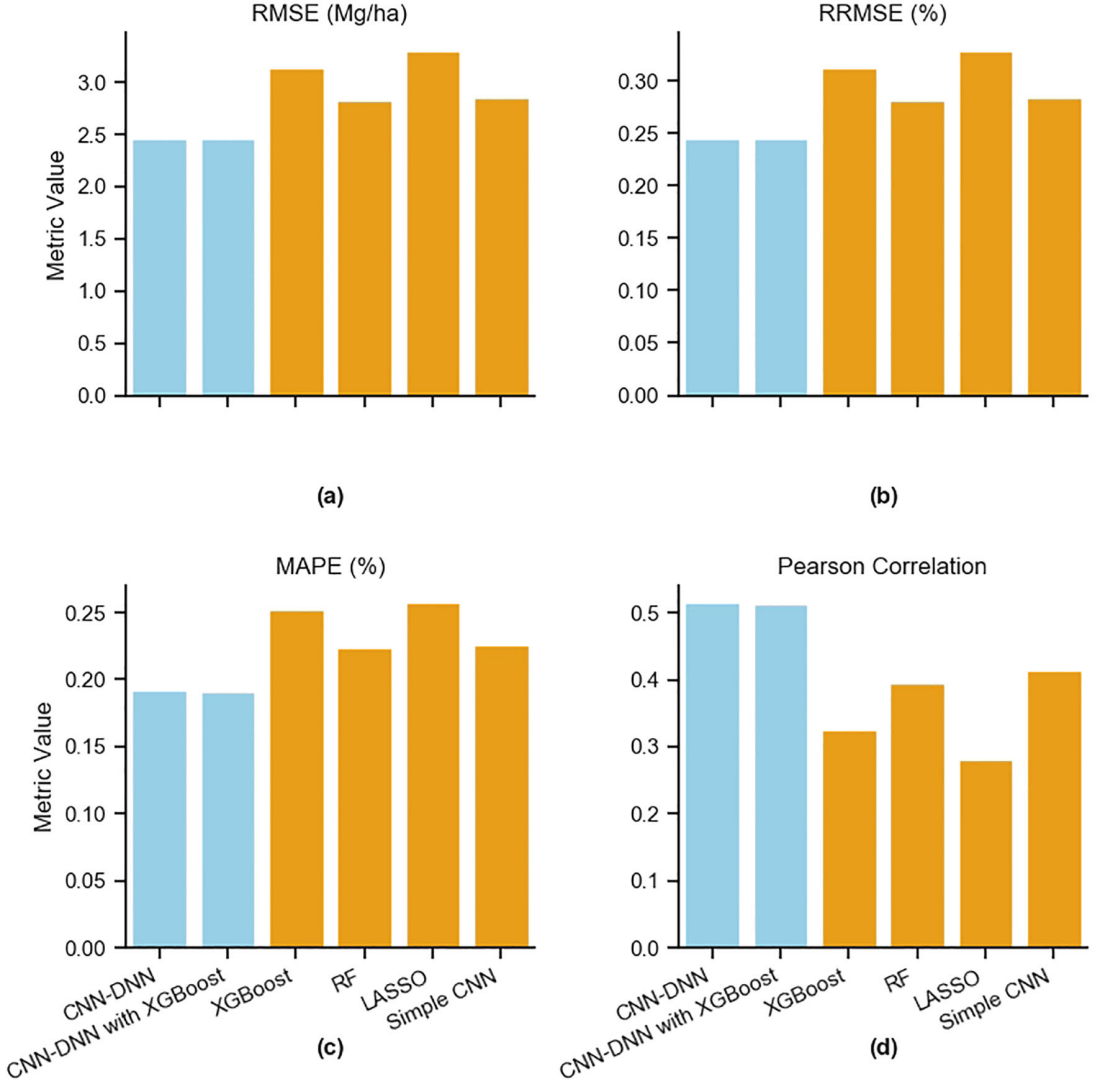
Comparison of prediction model performances. The proposed CNN-DNN model and base models are evaluated in terms of **(a)** RMSE, **(b)** RRMSE, **(c)** MAPE, and **(d)** Pearson Correlation.

All six models were trained on the training data and evaluated using the test data in terms of RMSE, RRMSE, MAPE, and Pearson Correlation (**Figure 14**; **Supplementary Material Table 3**). Comparing all metrics revealed that Lasso had the lowest performance (RMSE: 3.31 Mg/ha; RRMSE: 33%; MAPE: 22%; Correlation: 0.28), followed by XGBoost trained on the entire data (RMSE of 3.14 Mg/ha, RRMSE of 31%, MAPE 25%, Correlation: 0.32), the simple CNN model (RMSE: 2.85 Mg/ha; RRMSE: 28%; MAPE: 23%; Correlation: 0.41), and the RF model (RMSE: 2.82 Mg/ha; RRMSE: 28%; MAPE: 22%; Correlation: 0.39), The proposed CNN-DNN had an RMSE of 2.46 Mg/ha and the CNN-DNN model ensembled with XGBoost (trained on yield features) had an RMSE of 2.45, while other matrics having similar values ((RRMSE: 24%; MAPE: 19%; Correlation: 0.51) (**Figure 14**). This indicates that the proposed CNN-DNN and CNN-DNN ensembled with XGBoost outperform other prediction models. It also highlights that due to the intricate relationship between genotype, environment, and management, a simple CNN with all these inputs fed together has lower predictability. The benefit of using modular blocks for different types of inputs is evident.

The dataset utilized in this study originates from the 2022 Genomes to Fields (G2F) competition (Lima et al., 2023; The Genomes To Fields Initiative, 2023). A comparative evaluation of the proposed CNN-DNN and CNN-DNN combined with XGBoost models against other submissions using the same dataset indicates that our model achieved an RMSE of 2.46 Mg/ha. This result falls within the range of the top 10 highest-performing models (Washburn et al., 2024), demonstrating that the proposed approach offers competitive predictive performance while additionally providing enhanced interpretability.

## 4 Discussion

Uncovering the interactions between G×E×M factors and crop yield can enhance productivity. In this study, an exploratory analysis was conducted to identify key factors influencing crop yield under various scenarios, such as drought, standard conditions, disease trials, and irrigation. Results showed significant yield variation based on hybrid-location combinations, and planting dates were found to impact yield. Additionally, an empirical rule-based tool was developed for high-yield hybrid classification across different scenarios. Finally, a multimodal CNN-DNN model, ensembled with XGBoost, was created to predict yield considering G×E×M interactions. This section summarizes the key findings of this study.

Selecting informative loci from genotype data is crucial for yield prediction model performance. Using the entire genotype dataset can lead to a curse of dimensionality, so choosing an appropriate subset is essential. Iterative analysis revealed that selecting around 300 loci improves prediction accuracy, given the training data size. Systematic selection of these loci, as opposed to random selection, enhances prediction because random selection may include less informative loci. The proposed method for locus selection demonstrated better yield prediction results.

In addition to using appropriate feature selection methods, the imputation technique is important, particularly when dealing with datasets with many missing values. Given the locational dependency of the observations, location-based imputation significantly aided the prediction model’s learning. However, observations with numerous missing values were excluded to prevent the model from learning from synthetic data. Deciding the extent of imputation and which observations to exclude was a key aspect of the data preprocessing stage.

The analysis of model architecture showed that a multimodal CNN-DNN model outperforms a single-modal CNN-DNN model in terms of predictability. In single-modal models, temporal and spatial dependencies are often lost when using various data sources for prediction. Notably, for weather data, a 2D CNN block was more effective than a 1D CNN or LSTM block in feature extraction and model prediction. Converting the 16 distinct weather features from week 1 to week 48 into a 3D array, with the y-axis representing the week number and the z-axis representing each weather feature, preserved temporal dependencies and improved the model’s performance.

Exploring the model prediction results, it was observed that the mean yield for state-hybrid combinations is well correlated with the observed yield for combinations that appeared at least twice during the training data (Pearson correlation: 0.78 for training data; 0.35 for test data). However, for new hybrid-state combinations, this feature had less predictability, indicating that crop yield can vary widely due to G×E interactions.

An evaluation of model performance across various treatments revealed that the model had the best predictability for irrigation (RMSE 2.31 Mg/ha) and standard treatment (RMSE 2.36 Mg/ha), followed by dryland (RMSE 2.48 Mg/ha) and drought (RMSE 2.85 Mg/ha). The highest error was observed in the late planting scenario (RMSE 3.91 Mg/ha), likely due to the training data containing only synthetic observations for this scenario. This suggests that the model performs better for treatments or scenarios included in the training data; however, it has limitations in predicting yield for new management practices it has not encountered. This limitation could be addressed by expanding the training dataset to include a broader range of management practices. Additionally, incorporating satellite data to capture crop growth stages may improve the model’s ability to predict outcomes under extreme conditions.

**Figure 15:**
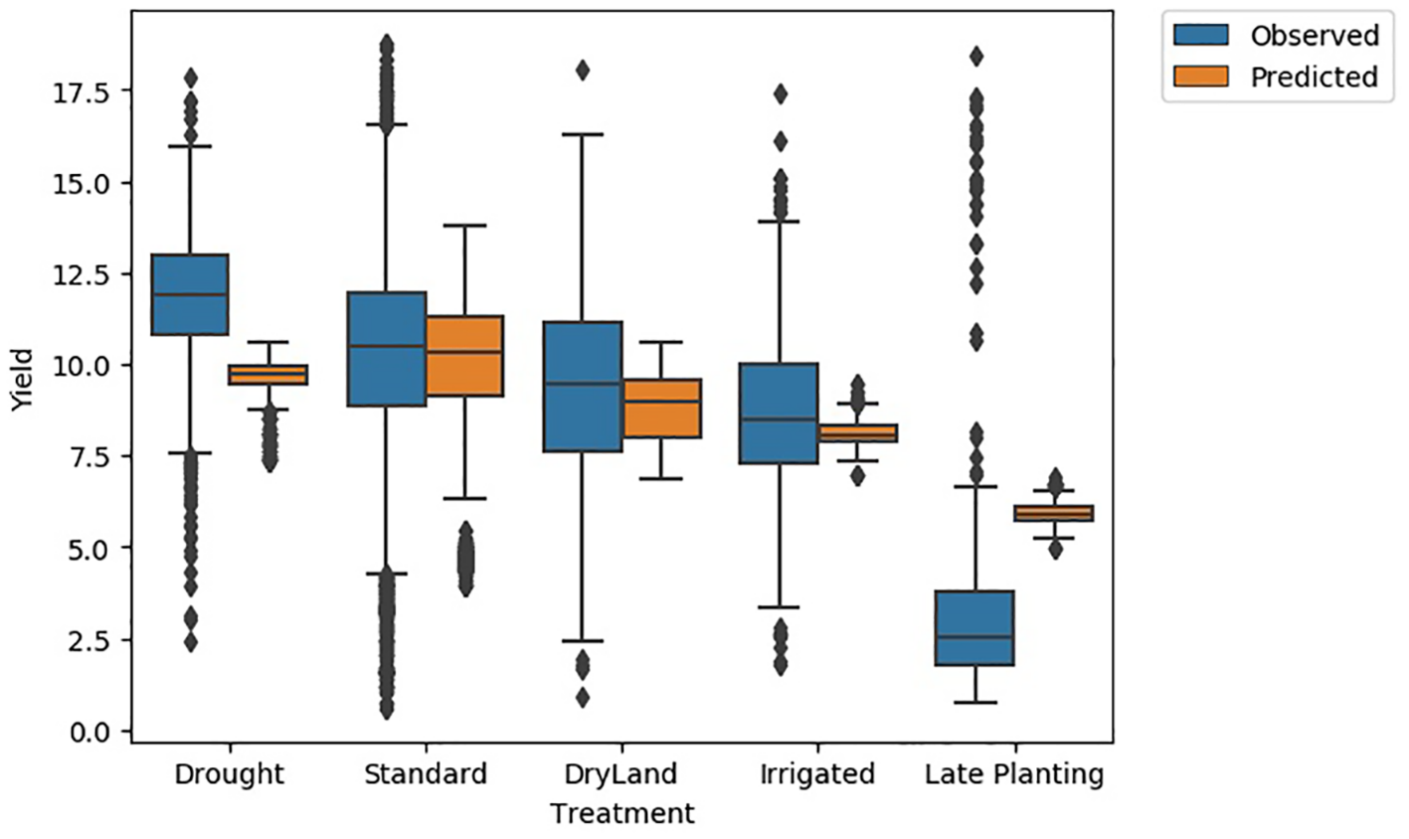
Evaluation of model performance for different treatment types.

Examining the observed and predicted yield distributions for various scenarios revealed that the distributions for the standard treatment were nearly identical (**Figure 15**). For drought, the observed yield distribution was higher (25th percentile at 11 Mg/ha, mean at 12 Mg/ha, and 75th percentile at 14.5 Mg/ha) compared to the predicted distribution (25th percentile at 9 Mg/ha, mean at 9.5 Mg/ha, and 75th percentile at 10 Mg/ha). Conversely, for late planting, the predicted yield distribution (25th percentile at 5 Mg/ha, mean at 5.5 Mg/ha, and 75th percentile at 6 Mg/ha) was higher than the observed distribution (25th percentile at 2 Mg/ha, mean at 2.5 Mg/ha, and 75th percentile at 4 Mg/ha). This discrepancy is due to the lack of real training data for late planting and the presence of both high and low-yield scenarios for drought in the training data. For other treatments, the predicted and observed yield distributions overlap.

## 5 Conclusion

This research investigated the factors influencing agricultural productivity, proposed a hybrid selection tool to improve yields under extreme weather conditions or specific locations, and developed a yield prediction model. The exploratory data analysis highlighted the variations in yield related to genotype, environment, and management practices. Particularly, yield varied significantly based on location and hybrid interaction, with stable historical data providing good predictability for future outcomes. A prediction model incorporating genotype, environment, and management interactions (G x E x M) was developed using a multimodal CNN-DNN model. This model demonstrated its effectiveness in predicting field-level productivity across various management practices, weather conditions, locations, and hybrids. Future research could focus on estimating other phenotypic traits at different crop cycle phases and incorporating spatial properties into the hybrid identification tool for various scenarios. To better address unforeseen conditions, in addition to increasing training data, crop simulation studies grounded in plant science and physiology could also be incorporated into the prediction framework.

## Data Availability

Publicly available datasets were analyzed in this study. This data can be found here: https://datacommons.cyverse.org/browse/iplant/home/shared/commons_repo/curated/GenomesToFields_G2F_data_2022.

## References

[B1] Anapalli S. S. Ma L. Nielsen D. C. Vigil M. F. Ahuja L. R. (2005). Simulating planting date effects on corn production using RZWQM and CERES-maize models. Agron. J. 97, 58–71. doi: 10.2134/agronj2005.0058

[B2] Bali N. Singla A. (2021). Emerging trends in machine learning to predict crop yield and study its influential factors: A survey. Arch. Comput. Methods Eng. 29, 95–112. doi: 10.1007/S11831-021-09569-8

[B3] Baltrusaitis T. Ahuja C. Morency L. P. (2019). Multimodal machine learning: A survey and taxonomy. IEEE Trans. Pattern Anal. Mach. Intell. 41, 423–443. doi: 10.1109/TPAMI.2018.2798607, PMID: 29994351

[B4] Beres B. L. Hatfield J. L. Kirkegaard J. A. Eigenbrode S. D. Pan W. L. Lollato R. P.. (2020). Toward a better understanding of genotype × Environment × Management interactions—A global wheat initiative agronomic research strategy. Front. Plant Sci. 11. doi: 10.3389/FPLS.2020.00828/BIBTEX, PMID: 32612624 PMC7308648

[B5] Bollero G. A. Bullock D. G. Hollinger S. E. (1996). Soil temperature and planting date effects on corn yield, leaf area, and plant development. Agron. J. 88, 385–390. doi: 10.2134/agronj1996.00021962008800030005x

[B6] Breiman L. (2001). Random forests. Mach. Learn. 45, 5–32. doi: 10.1023/A:1010933404324

[B7] Brown G. (2017). Ensemble Learning (Boston, MA: Springer). doi: 10.1007/978-1-4899-7687-1_252

[B8] Buitinck L. Louppe G. Blondel M. Pedregosa F. Müller A. C. Grisel O.. (2013). API design for machine learning software: experiences from the scikit-learn project. Available online at: https://arxiv.org/abs/1309.0238v1 (Accessed June 24, 2024).

[B9] Chen T. Guestrin C. (2016). “XGBoost: A scalable tree boosting system,” in *Proceedings of the ACM SIGKDD International Conference on Knowledge Discovery and Data Mining* 13-17-August-2016, 785–794. doi: 10.1145/2939672.2939785

[B10] Clevert D. A. Unterthiner T. Hochreiter S. (2015). “Fast and accurate deep network learning by exponential linear units (ELUs),” in 4th International Conference on Learning Representations, ICLR 2016 - Conference Track Proceedings. Available at: https://arxiv.org/abs/1511.07289v5.

[B11] Crossa J. Martini J. W. R. Gianola D. Pérez-Rodríguez P. Jarquin D. Juliana P.. (2019). Deep kernel and deep learning for genome-based prediction of single traits in multienvironment breeding trials. Front. Genet. 10. doi: 10.3389/FGENE.2019.01168/BIBTEX, PMID: 31921277 PMC6913188

[B12] Cunha R. L. F. Silva B. Netto M. A. S. (2018). “A scalable machine learning system for pre-season agriculture yield forecast,” in *Proceedings - IEEE 14th International Conference on eScience, e-Science* 2018, 423–430. doi: 10.1109/ESCIENCE.2018.00131

[B13] Cutler D. R. Edwards T. C. Beard K. H. Cutler A. Hess K. T. Gibson J.. (2007). RANDOM FORESTS FOR CLASSIFICATION IN ECOLOGY. Ecology 88, 2783–2792. doi: 10.1890/07-0539.1, PMID: 18051647

[B14] Danilevicz M. F. Gill M. Anderson R. Batley J. Bennamoun M. Bayer P. E.. (2022). Plant genotype to phenotype prediction using machine learning. Front. Genet. 13. doi: 10.3389/FGENE.2022.822173/BIBTEX PMC9159391 35664329

[B15] Darby H. M. Lauer J. G. (2002). Planting date and hybrid influence on corn forage yield and quality. Agron. J. 94, 281–289. doi: 10.2134/agronj2002.0281

[B16] Dhaliwal D. S. Williams M. M. (2024). Sweet corn yield prediction using machine learning models and field-level data. Precis. Agric. 25, 51–64. doi: 10.1007/S11119-023-10057-1/FIGURES/3

[B17] Dobermann A. Ping J. L. Adamchuk V. I. Simbahan G. C. Ferguson R. B. (2003). Classification of crop yield variability in irrigated production fields. Agron. J. 95, 1105–1120. doi: 10.2134/AGRONJ2003.1105

[B18] Dobor L. Barcza Z. Hlásny T. Árendás T. Spitkó T. Fodor N. (2016). Crop planting date matters: Estimation methods and effect on future yields. Agric. For. Meteorol. 223, 103–115. doi: 10.1016/J.AGRFORMET.2016.03.023

[B19] Elavarasan D. Durairaj Vincent P. M. (2020). Crop yield prediction using deep reinforcement learning model for sustainable agrarian applications. IEEE Access 8, 86886–86901. doi: 10.1109/ACCESS.2020.2992480

[B20] Everingham Y. Sexton J. Skocaj D. Inman-Bamber G. (2016). Accurate prediction of sugarcane yield using a random forest algorithm. Agron. Sustain. Dev. 36, 1–9. doi: 10.1007/S13593-016-0364-Z/FIGURES/3

[B21] Fernandes I. K. Vieira C. C. Dias K. O. G. Fernandes S. B. (2024). Using machine learning to integrate genetic and environmental data to model genotype-by-environment interactions. bioRxiv. doi: 10.1101/2024.02.08.579534. 2024.02.08.579534.

[B22] Filippi P. Jones E. J. Wimalathunge N. S. Somarathna P. D. S. N. Pozza L. E. Ugbaje S. U.. (2019). An approach to forecast grain crop yield using multi-layered, multi-farm data sets and machine learning. Precis. Agric. 20, 1015–1029. doi: 10.1007/S11119-018-09628-4/FIGURES/5

[B23] Gambin B. L. Coyos T. Di Mauro G. Borrás L. Garibaldi L. A. (2016). Exploring genotype, management, and environmental variables influencing grain yield of late-sown maize in central Argentina. Agric. Syst. 146, 11–19. doi: 10.1016/J.AGSY.2016.03.011

[B24] Genomes to Fields (2023). Genomes to fields 2022 dataset. CyVerse. Data Commons. doi: 10.25739/3d3g-pe51

[B25] Grinberg N. F. Orhobor O. I. King R. D. (2020). An evaluation of machine-learning for predicting phenotype: studies in yeast, rice, and wheat. Mach. Learn. 109, 251–277. doi: 10.1007/S10994-019-05848-5/FIGURES/9, PMID: 32174648 PMC7048706

[B26] Hankinson M. W. Lindsey L. E. Culman S. W. (2015). Effect of planting date and starter fertilizer on soybean grain yield. Crop. Forage. Turfgrass Manage. 1, 1–6. doi: 10.2134/cftm2015.0178

[B27] Haque F. F. Abdelgawad A. Yanambaka V. P. Yelamarthi K. (2020). “Crop yield prediction using deep neural network,” in IEEE World Forum on Internet of Things, WF-IoT 2020 - Symposium Proceedings. doi: 10.1109/WF-IOT48130.2020.9221298, PMID:

[B28] Heffner E. L. Sorrells M. E. Jannink J. L. (2009). Genomic Selection for crop improvement. Crop Sci. 49, 1–12. doi: 10.2135/CROPSCI2008.08.0512

[B29] James G. Witten D. Hastie T. Tibshirani R. (2013). An introduction to statistical learning. 2nd ed. (Newyork: Springer). Available online at: http://www.springer.com/series/417.

[B30] Kaiser D. E. Coulter J. A. Vetsch J. A. (2016). Corn hybrid response to in-furrow starter fertilizer as affected by planting date. Agron. J. 108, 2493–2501. doi: 10.2134/agronj2016.02.0124

[B31] Khaki S. Wang L. (2019). Crop yield prediction using deep neural networks. Front. Plant Sci. 10. doi: 10.3389/FPLS.2019.00621/BIBTEX PMC6540942 31191564

[B32] Khaki S. Wang L. Archontoulis S. V. (2020). A CNN-RNN framework for crop yield prediction. Front. Plant Sci. 10. doi: 10.3389/FPLS.2019.01750/BIBTEX, PMID: 32038699 PMC6993602

[B33] Kolipaka V. R. R. Namburu A. (2024). An automatic crop yield prediction framework designed with two-stage classifiers: a meta-heuristic approach. Multimed. Tools Appl. 83, 28969–28992. doi: 10.1007/S11042-023-16612-2/METRICS

[B34] Kouadio L. Deo R. C. Byrareddy V. Adamowski J. F. Mushtaq S. Phuong Nguyen V. (2018). Artificial intelligence approach for the prediction of Robusta coffee yield using soil fertility properties. Comput. Electron. Agric. 155, 324–338. doi: 10.1016/J.COMPAG.2018.10.014

[B35] Lauer J. G. Carter P. R. Wood T. M. Diezel G. Wiersma D. W. Rand R. E.. (1999). Corn hybrid response to planting date in the northern corn belt. Agron. J. 91, 834–839. doi: 10.2134/agronj1999.915834x

[B36] Lima D. C. Washburn J. D. Varela J. I. Chen Q. Gage J. L. Romay M. C.. (2023). Genomes to fields 2022 maize genotype by environment prediction competition. BMC Res. Notes 16, 1–3. doi: 10.1186/S13104-023-06421-Z/TABLES/1 37461058 PMC10353085

[B37] Lopez-Cruz M. Aguate F. M. Washburn J. D. De Leon N. Kaeppler S. M. Lima C.. (2023). Leveraging data from the Genomes-to-Fields Initiative to investigate genotype-by-environment interactions in maize in North America. Nat. Commun. 14, 1–14. doi: 10.1038/s41467-023-42687-4, PMID: 37903778 PMC10616096

[B38] Ma W. Qiu Z. Song J. Li J. Cheng Q. Zhai J.. (2018). A deep convolutional neural network approach for predicting phenotypes from genotypes. Planta 248, 1307–1318. doi: 10.1007/S00425-018-2976-9/METRICS 30101399

[B39] Maestrini B. Basso B. (2018). Predicting spatial patterns of within-field crop yield variability. Field Crops Res. 219, 106–112. doi: 10.1016/J.FCR.2018.01.028

[B40] Maimaitijiang M. Sagan V. Sidike P. Hartling S. Esposito F. Fritschi F. B. (2020). Soybean yield prediction from UAV using multimodal data fusion and deep learning. Remote Sens. Environ. 237, 111599. doi: 10.1016/J.RSE.2019.111599

[B41] Masud Rana M. Belal Hossain M. Kumar Roy T. Shultana R. Rokebul Hasan M. Aminun Naher U.. (2024). Response of Yield and Agronomic Output of Bangabandhu dhan100 under Varying Sowing Window in Cold Prone Rangpur Region. Indian J. Agric. Res. 58, 259–265. doi: 10.18805/IJARe.AF-796

[B42] Medsker L. R. Jain L. (2001). Recurrent neural networks. Design and Applications. 5 (64-67), 2.

[B43] Mia M. S. Tanabe R. Habibi L. N. Hashimoto N. Homma K. Maki M.. (2023). Multimodal deep learning for rice yield prediction using UAV-based multispectral imagery and weather data. Remote Sens. (Basel). 15, 2511. doi: 10.3390/RS15102511/S1

[B44] Mir R. R. Reynolds M. Pinto F. Khan M. A. Bhat M. A. (2019). High-throughput phenotyping for crop improvement in the genomics era. Plant Sci. 282, 60–72. doi: 10.1016/J.PLANTSCI.2019.01.007, PMID: 31003612

[B45] Monroe J. G. Arciniegas J. P. Moreno J. L. Sánchez F. Sierra S. Valdes S.. (2020). The lowest hanging fruit: Beneficial gene knockouts in past, present, and future crop evolution. Curr. Plant Biol. 24, 100185. doi: 10.1016/J.CPB.2020.100185

[B46] Montesinos-López O. A. Montesinos-López A. Crossa J. Gianola D. Hernández-Suárez C. M. Martín-Vallejo J. (2018b). Multi-trait, multi-environment deep learning modeling for genomic-enabled prediction of plant traits. G3 Genes|Genomes|Genet. 8, 3829–3840. doi: 10.1534/G3.118.200728, PMID: 30291108 PMC6288830

[B47] Montesinos-López A. Montesinos-López O. A. Gianola D. Crossa J. Hernández-Suárez C. M. (2018a). Multi-environment genomic prediction of plant traits using deep learners with dense architecture. G3 Genes|Genomes|Genet. 8, 3813–3828. doi: 10.1534/G3.118.200740, PMID: 30291107 PMC6288841

[B48] Moseley D. Reis A. Gentimis T. Campos P. Copes J. Netterville M.. (2024). Soybean planting dates and maturity groups: Maximizing yield potential and decreasing risk in Louisiana. Agron. J. 116 (5), 2174–2185. doi: 10.1002/AGJ2.21626

[B49] Mrubata K. Nciizah A. D. Muchaonyerwa P. (2024). Planting date and tillage effects on yield and nutrient uptake of two sorghum cultivars grown in sub-humid and semi-arid regions in South Africa. Front. Agron. 6. doi: 10.3389/FAGRO.2024.1388823/BIBTEX

[B50] Nevavuori P. Narra N. Lipping T. (2019). Crop yield prediction with deep convolutional neural networks. Comput. Electron. Agric. 163, 104859. doi: 10.1016/J.COMPAG.2019.104859

[B51] Nuccio M. L. Paul M. Bate N. J. Cohn J. Cutler S. R. (2018). Where are the drought tolerant crops? An assessment of more than two decades of plant biotechnology effort in crop improvement. Plant Sci. 273, 110–119. doi: 10.1016/J.PLANTSCI.2018.01.020, PMID: 29907303

[B52] Oakey H. Cullis B. Thompson R. Comadran J. Halpin C. Waugh R. (2016). Genomic selection in multi-environment crop trials. G3.: Genes. Genomes. Genet. 6, 1313–1326. doi: 10.1534/G3.116.027524/-/DC1 PMC4856083 26976443

[B53] Oikonomidis A. Catal C. Kassahun A. (2022). Hybrid deep learning-based models for crop yield prediction. Appl. Artif. Intell. 36 (1), 2031822. doi: 10.1080/08839514.2022.2031823/FORMAT/EPUB

[B54] Paudel D. Boogaard H. de Wit A. van der Velde M. Claverie M. Nisini L.. (2022). Machine learning for regional crop yield forecasting in Europe. Field Crops Res. 276, 108377. doi: 10.1016/J.FCR.2021.108377

[B55] Sajid S. S. Shahhosseini M. Huber I. Hu G. Archontoulis S. V. (2022). County-scale crop yield prediction by integrating crop simulation with machine learning models. Front. Plant Sci. 13. doi: 10.3389/FPLS.2022.1000224/BIBTEX, PMID: 36518505 PMC9742473

[B56] Sarzaeim P. Muñoz-Arriola F. (2024). A method to estimate climate drivers of maize yield predictability leveraging genetic-by-environment interactions in the US and Canada. Agronomy 14, 733. doi: 10.3390/AGRONOMY14040733

[B57] Scheben A. Wolter F. Batley J. Puchta H. Edwards D. (2017). Towards CRISPR/Cas crops – bringing together genomics and genome editing. New Phytol. 216, 682–698. doi: 10.1111/NPH.14702, PMID: 28762506

[B58] Shahhosseini M. Hu G. Archontoulis S. V. (2020). Forecasting corn yield with machine learning ensembles. Front. Plant Sci. 11. doi: 10.3389/fpls.2020.01120, PMID: 32849688 PMC7411227

[B59] Shahhosseini M. Hu G. Huber I. Archontoulis S. V. (2021a). Coupling machine learning and crop modeling improves crop yield prediction in the US Corn Belt. Sci. Rep. 11, 1–15. doi: 10.1038/s41598-020-80820-1, PMID: 33452349 PMC7810832

[B60] Shahhosseini M. Hu G. Khaki S. Archontoulis S. V. (2021b). Corn yield prediction with ensemble CNN-DNN. Front. Plant Sci. 12. doi: 10.3389/FPLS.2021.709008/FULL, PMID: 34408763 PMC8364956

[B61] Shook J. Gangopadhyay T. Wu L. Ganapathysubramanian B. Sarkar S. Singh A. K. (2021). Crop yield prediction integrating genotype and weather variables using deep learning. PloS One 16, e0252402. doi: 10.1371/JOURNAL.PONE.0252402, PMID: 34138872 PMC8211294

[B62] Swanson S. P. Wilhelm W. W. (1996). Planting date and residue rate effects on growth, partitioning, and yield of corn. Agron. J. 88, 205–210. doi: 10.2134/agronj1996.00021962008800020014x

[B63] The Genomes To Fields Initiative (2023). Available online at: https://www.genomes2fields.org/ (Accessed November 15, 2023).

[B64] Thorburn P. Dietzel R. Cammarano D. Ciampitti I. A. Long N. V. Assefa Y.. (2017). Maize yield and planting date relationship: A synthesis-analysis for US high-yielding contest-winner and field research data. Front. Plant Sci. 8, 2106. doi: 10.3389/fpls.2017.02106, PMID: 29312377 PMC5743010

[B65] Tibshiranit R. (1996). Regression shrinkage and selection via the lasso. J. R. Stat. Soc.: Ser. B. (Methodological). 58, 267–288. doi: 10.1111/J.2517-6161.1996.TB02080.X

[B66] Tsimba R. Edmeades G. O. Millner J. P. Kemp P. D. (2013). The effect of planting date on maize grain yields and yield components. Field Crops Res. 150, 135–144. doi: 10.1016/J.FCR.2013.05.028

[B67] Van Roekel R. J. Coulter J. A. (2011). Agronomic responses of corn to planting date and plant density. Agron. J. 103, 1414–1422. doi: 10.2134/agronj2011.0071

[B68] Wang A. X. Tran C. Desai N. Lobell D. Ermon S. (2018). “Deep transfer learning for crop yield prediction with remote sensing data,” in Proceedings of the 1st ACM SIGCAS Conference on Computing and Sustainable Societies, COMPASS, vol. 18. doi: 10.1145/3209811.3212707

[B69] Washburn J. D. Varela J. I. Xavier A. Chen Q. Ertl D. Gage J. L.. (2024). Global genotype by environment prediction competition reveals that diverse modeling strategies can deliver satisfactory maize yield estimates. bioRxiv. doi: 10.1101/2024.09.13.612969. 2024.09.13.612969., PMID: 39576009 PMC12054733

[B70] Williams M. M. (2006). Planting date influences critical period of weed control in sweet corn. Weed. Sci. 54, 928–933. doi: 10.1614/ws-06-005r.1

[B71] Yu H. Weng L. Wu S. He J. Yuan Y. Wang J.. (2024). Time-series field phenotyping of soybean growth analysis by combining multimodal deep learning and dynamic modeling. Plant Phenomics. 6, 0158. doi: 10.34133/PLANTPHENOMICS.0158/SUPPL_FILE/PLANTPHENOMICS.0158.F1.ZIP, PMID: 38524738 PMC10959008

