# Supplementary material for: Analyses of crop yield dynamics and the development of a multimodal neural network prediction model with G×E×M interactions
Source: Front Plant Sci. 2025 Jul 31;16:1537990. doi: 10.3389/fpls.2025.1537990 (PMC12350365; doi:10.3389/fpls.2025.1537990)
Supplement: Supplementary file 1 [file DataSheet1.docx]

**Supplementary Materials**


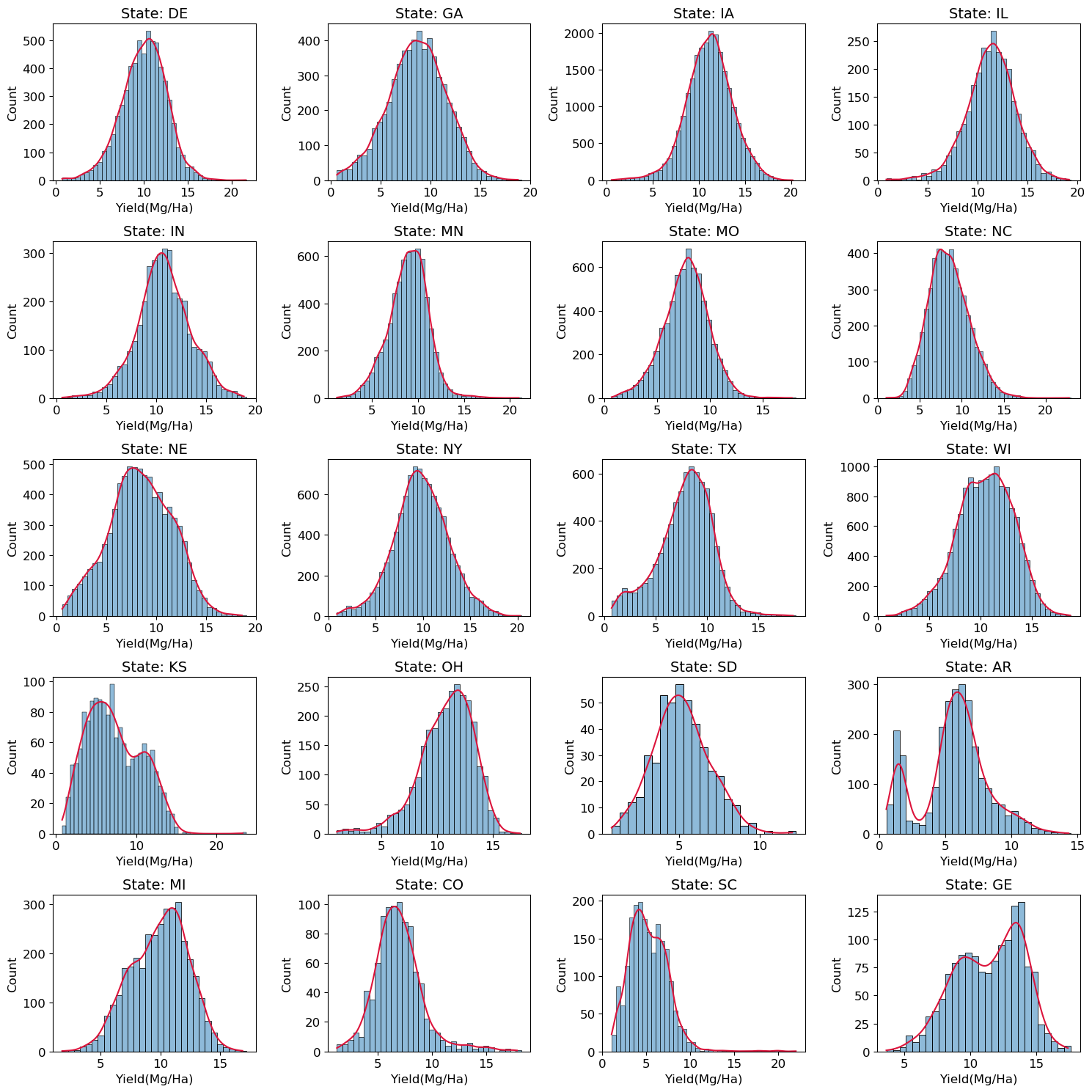


Figure 1: Yield distribution variation across states, with some states exhibiting bimodal distributions. While most states have unimodal distributions with different modes.


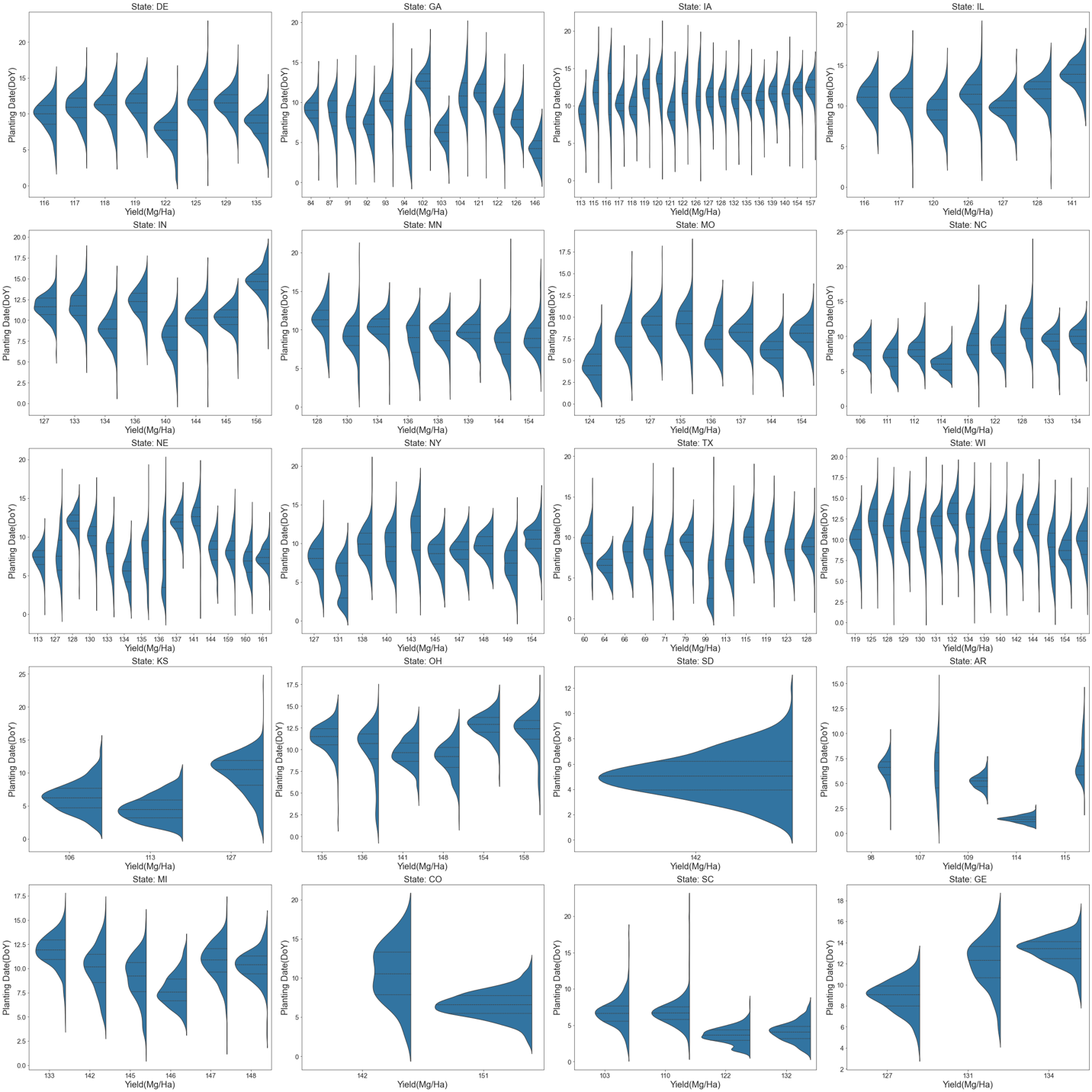


Figure 2: Yield distribution for different planting dates across states. For different planting dates, the yield distribution varies.


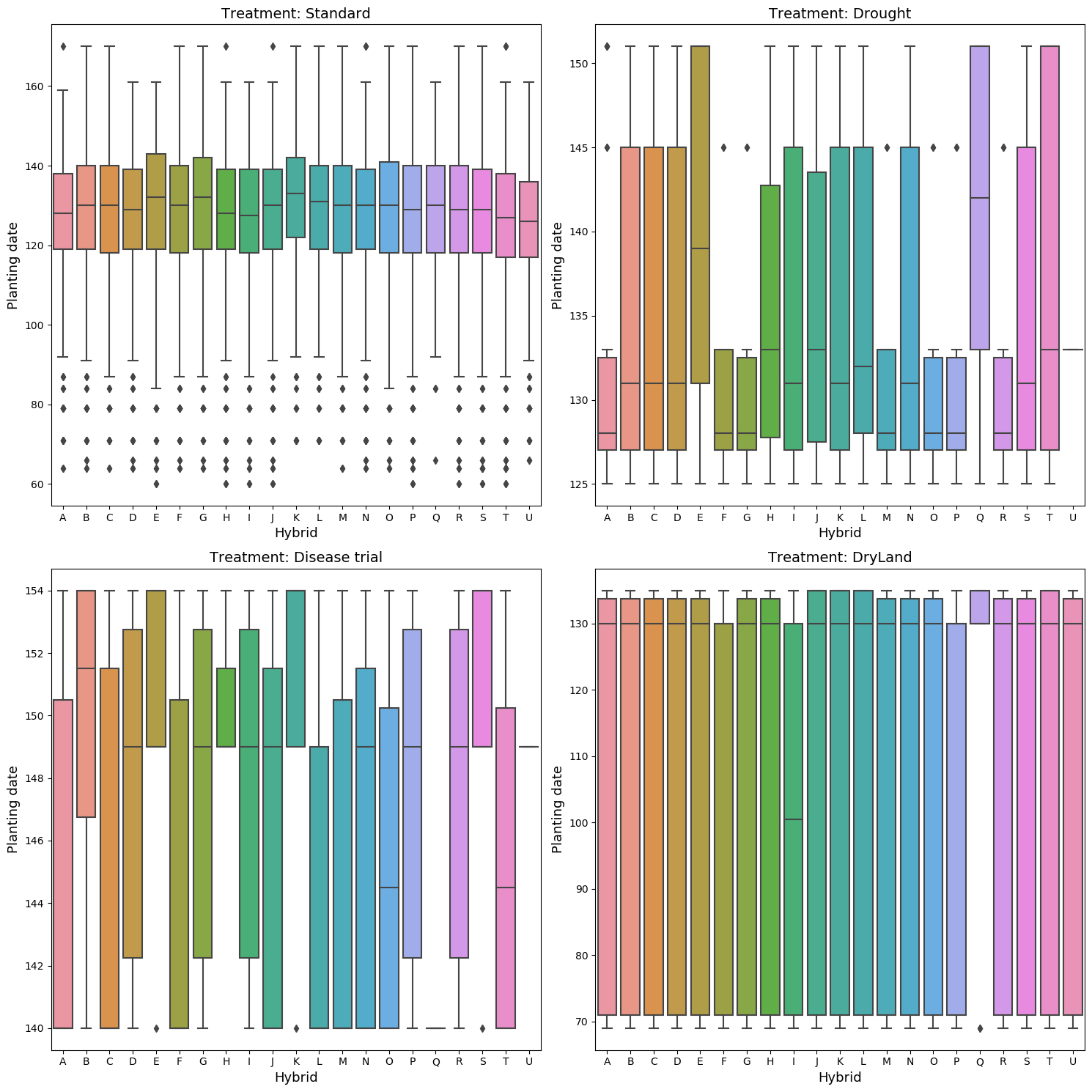


Figure 3: Identifying the planting window in various treatments for 21 hybrids planted during the late planting scenario in Texas
