# Supplementary material for: Analyses of crop yield dynamics and the development of a multimodal neural network prediction model with G×E×M interactions
Source: Front Plant Sci. 2025 Jul 31;16:1537990. doi: 10.3389/fpls.2025.1537990 (PMC12350365; doi:10.3389/fpls.2025.1537990)
Supplement: Supplementary file 2 [file Table1.docx]

Table 1: Detailed description of CNN-DNN model with layers, filters, and activation function

| Data | CNN Layer 1 | CNN Layer 2 | Average pool | Dense |
| --- | --- | --- | --- | --- |
| Weather | 2D,  Kernel size: (5x5)  Filters: 16  Activation: Elu | 2D,  Kernel size: (5x5)  Filters: 16  Activation: Elu | Pool size: (2x2)  Stride: (2x2) | Units:10  Activation: Elu |
| Soil | 1D,  Kernel size: 5  Filters: 16  Activation: Elu | 1D,  Kernel size: 3  Filters: 4  Activation: Elu | Pool size: 3  Stride: 3 | Units:5  Activation: Elu |
| Env soil for each soil features | 2D,  Kernel size: (5x5)  Filters: 16  Activation: Elu | 2D,  Kernel size: (3x3)  Filters:8  Activation: Elu | Pool size: (2x2)  Stride: (2x2) | Units:2  Activation: Elu |
| Env Phenological | 2D,  Kernel size: (5x5)  Filters: 10  Activation: Elu | 2D,  Kernel size: (3x3)  Filters: 5  Activation: Elu | Pool size: (2x2)  Stride: (2x2) | Units:3  Activation: Elu |
| Genotype | 1D,  Kernel size:5  Filters: 16  Activation: Elu | 1D,  Kernel size:3  Filters: 8  Activation: Elu | Pool size: 5  Stride: 5 | Units:8  Activation: Elu |
| Meta data | 1D,  Kernel size:5  Filters: 5  Activation: Elu | 1D,  Kernel size:1  Filters: 3  Activation: Elu | - | - |

Table 2: The selected 21 hybrid IDs represented by letter encoding

| Hybrid ID | Letter Code | Hybrid ID | Letter Code | Hybrid ID | Letter Code |
| --- | --- | --- | --- | --- | --- |
| '2369/LH123HT' | **A** | 'B73/MO17' | **H** | 'F42/OH43' | **O** |
| 'B14A/H95' | **B** | 'B73/PHM49' | **I** | 'LH74/PHN82' | **P** |
| 'B14A/MO17' | **C** | 'B73/PHN82' | **J** | 'PHB47/PHN82' | **Q** |
| 'B14A/OH43' | **D** | 'CG119/CG108' | **K** | 'PHG39/PHN82' | **R** |
| 'B37/H95' | **E** | 'CG44/CGR01' | **L** | 'PHW52/PHM49' | **S** |
| 'B37/MO17' | **F** | 'F42/H95' | **M** | 'PHW52/PHN82' | **T** |
| 'B37/OH43' | **G** | 'F42/MO17' | **N** | 'TX777/LH195' | **U** |

Table 3: Evaluation metric values for all prediction models

| **Model** | **Metric** | | | | | |
| --- | --- | --- | --- | --- | --- | --- |
|  | *R^2^ | RMSE | RRMSE | MAE | MAPE | Pearson correlation |
| CNN-DNN | 0.236 | 2.457 | 0.244 | 1.927 | 0.192 | 0.515 |
| CNN-DNN with XGBoost | 0.238 | 2.454 | 0.244 | 1.923 | 0.191 | 0.513 |
| XGBoost | -0.248 | 3.14 | 0.312 | 2.538 | 0.252 | 0.324 |
| RF | -0.009 | 2.823 | 0.281 | 2.247 | 0.223 | 0.395 |
| LASSO | -0.384 | 3.307 | 0.329 | 2.586 | 0.257 | 0.28 |
| Simple CNN | -0.032 | 2.857 | 0.284 | 2.272 | 0.226 | 0.414 |

*R^2^ is the coefficient of determination. For XGBoost, RF, LASSO, and Simple CNN, negative R^2^ indicates, these models perform poorly in predicting the average from the training data
